# Machine learning forecasts suggest a concentration paradox in international student mobility to the United Kingdom

**DOI:** 10.1038/s41467-026-77425-z

**Published:** 2026-09-17

**Authors:** Ruth Neville, Francisco Rowe, Emilio Zagheni

**Affiliations:** 1 Max Planck Institute for Demographic Research (MPIDR), Rostock, Germany; 2 Geographic Data Science Lab (GDSL), Department of Geography and Planning, University of Liverpool, Liverpool, UK; 3 Centre for Advanced Spatial Analysis (CASA), University College London, London, UK

**Keywords:** Geography, Education

## Abstract

International student mobility is central to higher education finance, soft power, and knowledge production, yet future demand is increasingly uncertain. We analyse Universities and Colleges Admissions Service records on successful international undergraduate applications to the United Kingdom from 86 origin countries between 2010 and 2024, linked to economic and demographic projections, to forecast demand to 2030. A Poisson-based gradient-boosted tree model, a machine-learning method that combines decision trees, is evaluated against autoregressive integrated moving average time-series models and negative binomial gravity models over an out-of-sample period spanning Brexit and the coronavirus pandemic. Forecast errors are lower for the machine-learning model, although gains are modest under structural shocks. Mainland China and Hong Kong, India, and the European Union are projected to account for more than half of international demand by 2030 despite stagnant or declining counts. This concentration paradox increases system vulnerability by intensifying reliance on a narrowing set of origins.

## Abstract (web summary)

Neville and colleagues compare machine-learning and traditional forecasts of United Kingdom international undergraduate demand, identifying a concentration paradox in which reliance on a few origins grows as application numbers stagnate or fall.

## Introduction

International student mobility (ISM) is a major component of high-skilled mobility and the international knowledge economy. In 2022, the global number of internationally mobile students was estimated at 6.9 million, representing an increase of 176% since 2002^1^. International students transfer knowledge, skills and social capital across borders, strengthening the academic and research capacity of host institutions while maintaining transnational connections with origin countries^2–4^. ISM can therefore contribute to human-capital development, brain circulation and wider economic, social and cultural exchange. However, these benefits are unevenly distributed. High-income countries remain the principal destinations, while lower-income origin countries may experience losses of highly skilled people and concerns over brain drain^5–7^.

International students have also become increasingly important to national strategies for economic growth, migration, diplomacy and soft power^8,9^. Student mobility occupies an ambiguous position between temporary educational movement and longer-term migration, with an estimated 15–35% of international students remaining in their host country after graduation^10^. International education consequently provides a potential pathway into high-skilled migration while also becoming entangled in political debates over immigration control^6,11^. This creates a recurring tension between policies intended to expand international student recruitment and wider efforts to restrict migration.

The geography of ISM reflects broader global inequalities and historical relationships. English-speaking destinations, particularly the United States, the United Kingdom (UK) and Australia, have emerged as dominant hosts, supported by the global reach of English-language education, colonial histories, cultural linkages and established international recruitment systems^8,12,13^. These three countries collectively host approximately 42% of international students, while Mainland China and India are among the largest origin countries^12^. Although high-income destination countries are often treated as the default frame of reference for international student flows^14^, this framing has been criticised for reproducing assumptions rooted in colonial histories and for obscuring substantial variation within both origin and destination countries^15^.

These patterns are shaped by economic development, institutional opportunities, historical ties and existing migration networks. Push–pull approaches emphasise differences between educational and economic opportunities in origin and destination countries^16,17^. Network and migration-systems perspectives further show how colonial relationships, shared languages and established migrant communities reduce the informational, financial and social costs of mobility^18–20^. Common language, particularly English, is therefore an important facilitator of entry into UK higher education institutions (HEIs)^21^.

Human-development dynamics also influence which countries send and receive students. Migration transition theory proposes that migration initially increases with development as rising resources, aspirations and capabilities enable more people to move, before stabilising or declining as countries become more attractive destinations themselves^22^. Applied to student mobility, this produces a U-shaped relationship in which countries initially send greater numbers of students abroad as they develop but subsequently expand their capacity to host students^23^. Previous work on the UK similarly shows that countries with higher levels of GDP per capita tend to send more students, although the magnitude and form of this relationship vary across origins^20^. Rapidly developing countries may therefore generate growing outbound demand in the medium term while simultaneously expanding domestic higher-education capacity in ways that eventually reduce overseas study.

Within this global system, the UK is a particularly important case. It is the second-leading destination for international students, attracting approximately 10–14% of the global market in recent years, and international students comprise around one-quarter of the UK student population^13^. They are central to the financial sustainability of UK HEIs, the wider economy, and the UK’s international influence. International students generated an estimated net economic benefit of more than £35 billion in 2021/22, while their tuition fees have become increasingly important for cross-subsidising teaching and research^24^. This dependence makes future changes in both the volume and composition of international student demand consequential for institutional planning and sector-wide stability.

The UK’s international student system has also experienced substantial policy and geopolitical disruption. Before Brexit, European Union (EU) countries collectively supplied the largest share of international undergraduate students, while Mainland China expanded rapidly to become the largest individual origin country^25^. The UK’s withdrawal from the EU fundamentally altered the conditions under which EU students could access UK higher education. From 2021, EU applicants lost home-fee status and access to the UK student-loan system and became subject to higher tuition fees and visa requirements^26^. Applications subsequently fell by approximately two-thirds, as demonstrated in previous work and other recent studies^27,28^.

At the same time, growth in applications from Mainland China has slowed, potentially reflecting changing geopolitical relations, demographic conditions and expanding domestic higher-education capacity. The UK government has sought to diversify recruitment by identifying countries including India, Indonesia, Saudi Arabia, Vietnam and Nigeria as priority markets^29^. However, this expansionary strategy exists alongside recurrent attempts to restrict migration, including limitations on student dependants, increased scrutiny of so-called ‘non-genuine’ applications, and debates over post-study work opportunities^30–32^. Reductions in sponsored study-visa applications during 2024 and projected financial deficits across many UK institutions have intensified concern about the resilience of the existing recruitment model^33^.

Attention to the composition of international demand is therefore as important as attention to its overall volume. Dependence on a small number of origin countries can generate financial and institutional vulnerability even when aggregate recruitment remains high. A policy change, economic crisis, geopolitical dispute or demographic shift affecting one major origin may have consequences across the UK higher-education system. Changes in the relative contribution of different origins may therefore reshape institutional exposure to external shocks without necessarily producing an immediate fall in total international student numbers.

Reliable forecasts are needed to anticipate these changes, but migration is among the most volatile demographic processes^34^. Economic crises, conflicts, pandemics and sudden policy reforms can generate abrupt departures from established trends and disrupt long-standing mobility corridors^35^. Student mobility is shaped by a dynamic combination of economic, political, demographic, social and institutional conditions, many of which interact nonlinearly and change rapidly. Although existing theories provide substantial insight into why ISM changes, they are less able to anticipate precisely when changes will occur or how large they will be.

These difficulties are visible in recent UK projections. Sector-level forecasts commonly use time-series approaches, including autoregressive integrated moving average (ARIMA) models, or gravity-type models that extrapolate from historical trends and incorporate economic, demographic and geographic characteristics^36–38^. Gravity models are theoretically grounded and widely used to explain human and student mobility^20,23,39^, while time-series models remain attractive because of their relative simplicity and interpretability. However, recent projections have often proved overly optimistic. In 2024, undergraduate applicant growth was forecast at 5.8%, whereas actual growth was only 1.3%, contributing to resource misallocation and unanticipated financial pressures across the sector^40^.

Machine-learning methods may provide an alternative by accommodating nonlinear relationships, interactions and heterogeneous trajectories across origin countries. Emerging research demonstrates their potential for student forecasting. Litmeyer and Hennemann^41^ use explainable machine-learning methods, including XGBoost, to forecast first-year enrolments at German universities and find that they can outperform gravity models while retaining information about variable importance. Yang et al.^42^ apply machine learning to outbound student mobility from Taiwan and report improved predictive performance relative to ARIMA and vector autoregression models. Nevertheless, academic research applying machine learning to ISM remains limited. Few studies use detailed administrative data to forecast mobility to a major destination country, and comparative assessments of machine-learning, time-series and theoretically informed gravity models remain rare.

In this work, we use Universities and Colleges Admissions Service administrative data on successful undergraduate applications from 86 origin countries between 2010 and 2024 to forecast international undergraduate demand to the UK through 2030. We compare a Poisson-based XGBoost model with autoregressive integrated moving average and negative binomial gravity benchmarks to address three questions: (1) how accurately can machine-learning methods predict recent trends in undergraduate international student applications to the UK relative to traditional forecasting approaches; (2) what future trajectories are projected for applications from key countries and regions of origin between 2025 and 2030; and (3) how do these trajectories reshape the concentration of UK international undergraduate demand? We show how applications from major origin countries can plateau or decline while their share of total demand continues to increase, producing a concentration paradox in which the UK’s dependence on a small number of origins grows even as inflows soften.

## Results

### Model performance

**Fig. 1:**
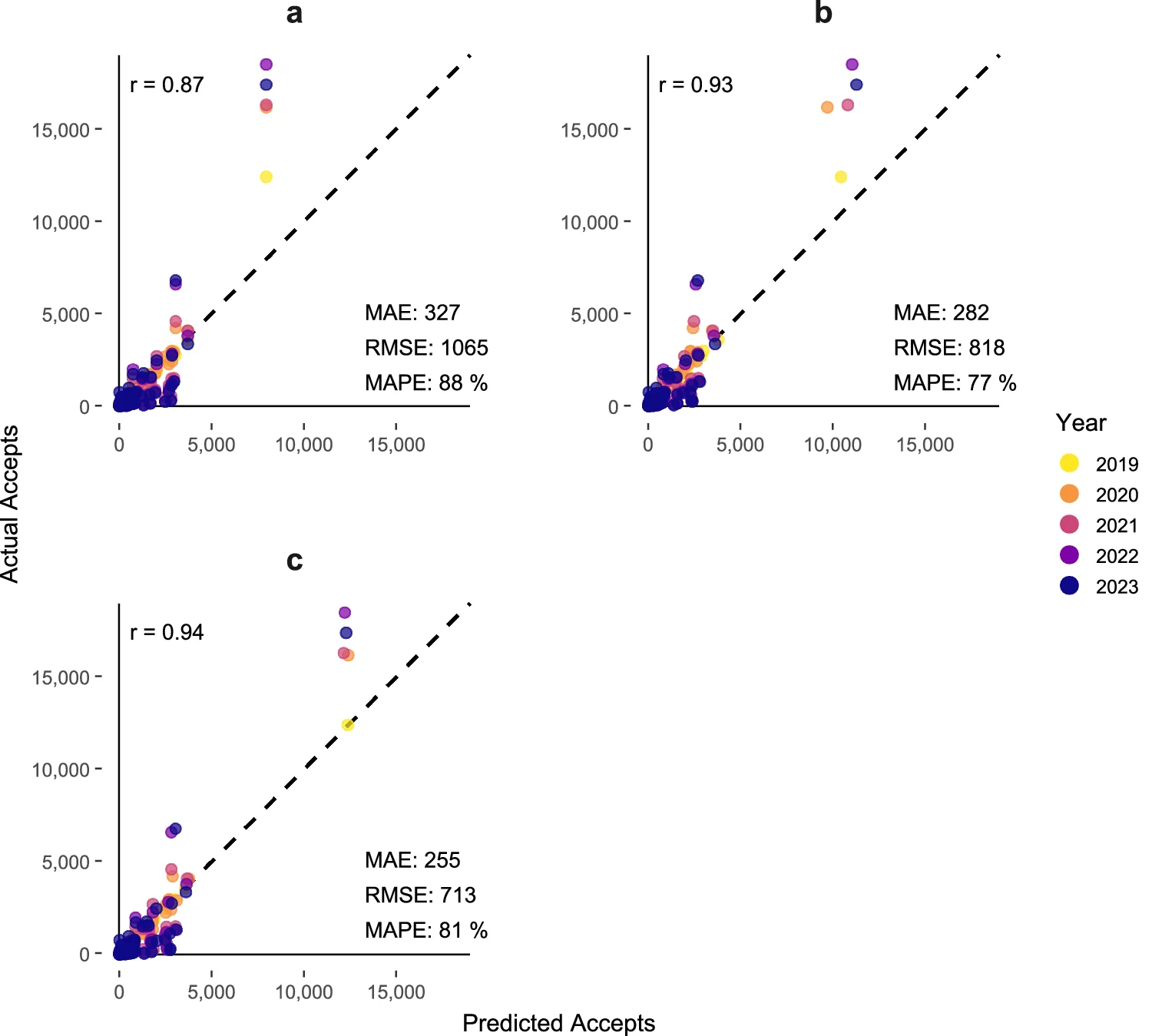
Model performance across the out-of-sample evaluation period. Predicted versus observed accepted undergraduate applications across origin countries and evaluation years for **a** autoregressive integrated moving average, **b** negative binomial gravity and **c** XGBoost models. Each point represents an origin country-year observation, colours indicate evaluation years, and dashed lines indicate the 45° line corresponding to perfect prediction. MAE denotes mean absolute error, RMSE denotes root mean squared error and MAPE denotes mean absolute percentage error.

Across the out-of-sample evaluation period (2019–2023), the XGBoost model demonstrates higher predictive accuracy than the ARIMA and negative binomial gravity benchmarks. Summary performance statistics across the three approaches are presented in Supplementary Table 3, and country-level out-of-sample predictions for 2019–2023 are shown in Fig. 1.

As shown in Fig. 1 and Supplementary Table 3, the XGBoost model achieves lower MAE and RMSE and stronger correlations with observed application counts, indicating improved capacity to capture non-linear interactions between economic, demographic and structural drivers. However, these improvements are incremental rather than transformative.

Although consistent, these performance gains remain modest. Predictive accuracy declines during periods affected by major policy developments, suggesting that uncertainty stems not only from methodological constraints but from structural characteristics of the system itself. This pattern is further illustrated in Supplementary Fig. 1, where deviations between predicted and observed values are most pronounced for origins affected by fee-status changes, visa policy adjustments or pandemic-related constraints. Sudden shifts in visa regulations, fee status and geopolitical relations introduce volatility that remains difficult to anticipate for traditional statistical and machine-learning models alike.

Taken together, these validation results indicate that XGBoost provides the most reliable basis for generating forecasts, while emphasising that projections remain conditional on the recent structural behaviour of the system. We therefore present forward-looking estimates below for the major sending regions that have shaped the UK’s international student intake over the past decade.

### Forecast trajectories for key origins

**Fig. 2:**
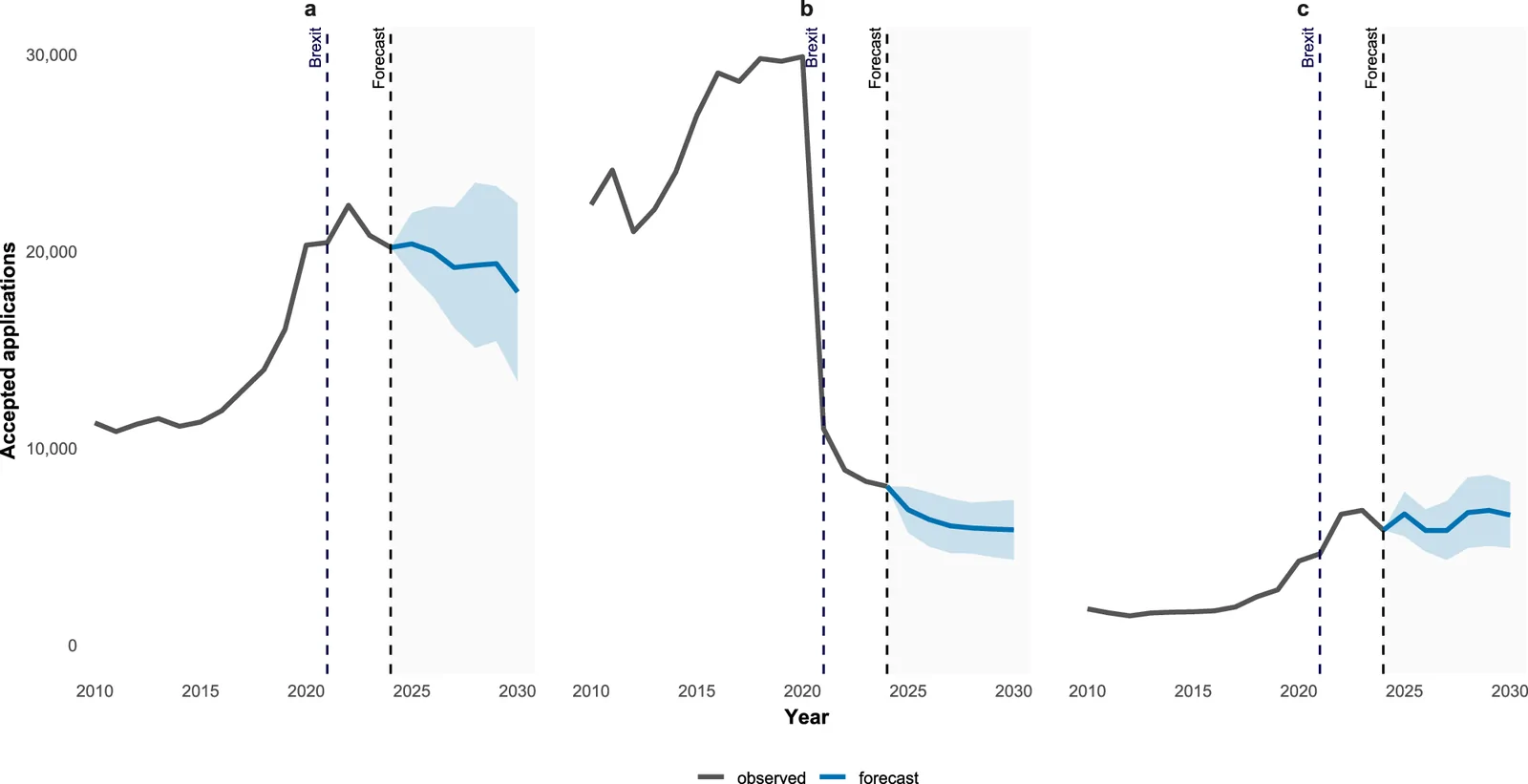
Forecast trajectories for the three principal origin groups. Forecasted undergraduate applications to United Kingdom universities from left to right: **a** Mainland China and Hong Kong, **b** the European Union and **c** India, 2010–2030. Black lines show observed annual acceptances for 2010–2024, blue lines show point forecasts for 2025–2030 and light-blue bands show 95% bootstrap prediction intervals. Vertical dashed lines mark the United Kingdom’s exit from the European Union and the boundary between the observed period ending in 2024 and the forecast period beginning in 2025.

**Fig. 3:**
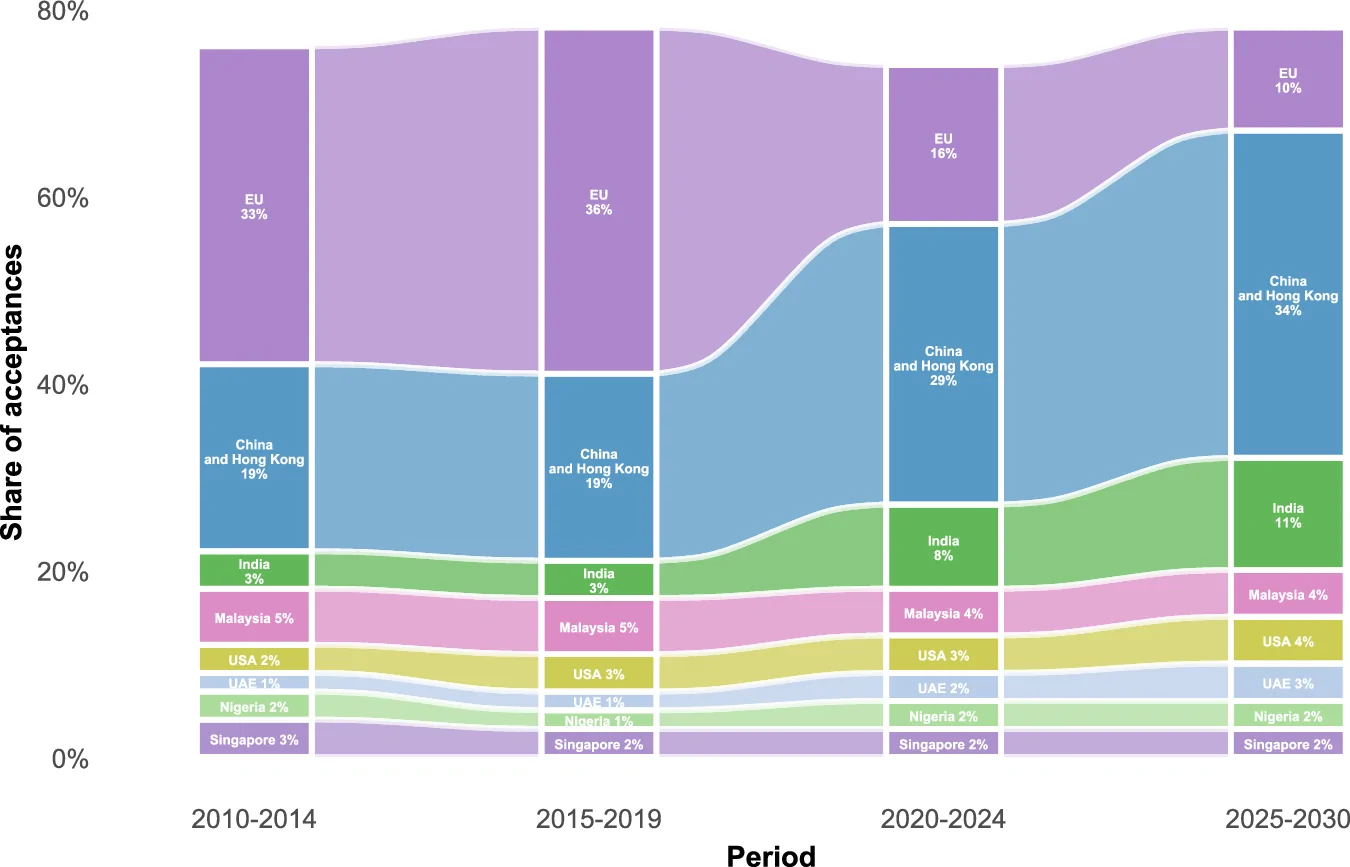
Changing composition of international student acceptances to the UK. This stacked alluvial plot shows the proportional share of undergraduate applications from the eight largest origin countries and regions between 2010 and 2030. The coloured bands represent the proportional contribution of each origin country or region over time. Based on historical data for 2010–2024 and forecasts to 2030, the figure highlights the post-Brexit decline in European Union applications and the growing reliance on India and Mainland China.

Figure 2 presents observed applications (2010–2024) and forecast trajectories (2025–2030) for the three major sending regions: Mainland China and Hong Kong, the European Union and India. Figure 3 summarises the changing proportional composition of the international applicant pool over the same period. These forecasts reflect concentration in international undergraduate demand, as captured by UCAS acceptance records.

Applications from European Union countries fell substantially after the UK’s withdrawal from the European Union and the implementation of new fee and visa regimes. Applications declined from approximately 30,000 in 2020 to around 9000 in 2024. The model projects a continued gradual decline to around 7000 by 2030, with narrow prediction intervals suggesting stability in the projected trajectory. This pattern reflects a structural regime change rather than cyclical fluctuation, with limited evidence of spontaneous recovery under current policy settings.

Proportionally, the European Union share of total international applications declines from around 36% in the pre-Brexit period to 16% in 2020–2024 and is projected to fall further to around 10% by 2025–2030.

Applications from India increased rapidly in the early 2010s and the immediate post-Brexit period, rising to around 6800 in 2023 before declining slightly in 2024. The model forecasts modest short-term recovery followed by stabilisation rather than continued steep growth. This suggests a shift from recruitment-led expansion to policy-sensitive stabilisation. Despite a plateau in application counts, India’s proportional share increases from approximately 8% in 2020–2024 to 11% in 2025–2030, shaped by declines and stagnation elsewhere in the system.

Mainland China and Hong Kong have remained the largest source of international undergraduate applications to the UK for much of the period. Applications decreased slightly from approximately 20,800 in 2023 to 20,200 in 2024. The model projects a gradual decline to roughly 18,000 applications by 2030. This dynamic aligns with evidence of growing domestic higher education capacity and shifting geopolitical relations.

Despite the projected moderation in absolute counts, the combined proportional share of Mainland China and Hong Kong is forecast to increase from around 29% in 2020–2024 to approximately 34% in 2025–2030. This increase is not driven by renewed expansion, but by sharper relative declines among other origins, reinforcing the broader concentration dynamics observed across the system.

Although presented here as a combined series for interpretability, predictions are generated separately for Mainland China and Hong Kong and aggregated post-estimation. Separate origin-level trajectories (Supplementary Fig. 2) indicate differences in scale and volatility between the two systems, with the decline in applications more pronounced for Mainland China than Hong Kong, although the combined trajectory remains dominated by Mainland China because of its substantially larger application volume.

### Changing composition and the concentration paradox

The results indicate a reconfiguration of the UK’s international undergraduate student base. Whereas the early 2010s were characterised by a diversified and expanding system, the late 2020s are projected to be defined by increased concentration among three major blocs that together account for more than half of projected international applications.

This configuration constitutes a concentration paradox: market share increases for some origins even as their application counts stabilise or decline. From a migration-systems perspective, this suggests that future uncertainty is increasingly concentrated rather than evenly distributed. As a small number of corridors account for a growing share of inflows, the stability of the system becomes more reliant on the political, economic and educational dynamics of these specific origins. This highlights that the risks inherent in forecasting are less a function of model specification and more a consequence of the structural properties and geopolitical contours shaping ISM.

## Discussion

This study provides a comprehensive application of machine learning methods to forecasting international student applications to the UK. By combining high-frequency administrative data from UCAS with economic, demographic and geographical covariates, and evaluating performance against traditional forecasting approaches, it offers methodological and substantive contributions to both ISM and migration forecasting literature.

Methodologically, the analysis demonstrated that Poisson-based XGBoost models can improve upon widely used ARIMA and gravity-type models in predicting recent mobility and ISM specifically. The machine learning approach captures non-linear relationships and interactions, such as how changes in GDP per capita and population interact with lagged application counts, in ways that are difficult to specify in parametric models. These gains, while incremental, are consistent across error metrics and robust in an out-of-sample evaluation period that includes substantial disruption from Brexit and COVID-19.

At the same time, the results reinforce the limits of purely data-driven forecasting when faced with structural shocks and policy discontinuities. The model performs less well for origins where policy interventions or conflicts generated abrupt changes in mobility that were not present in the training data. This is not unique to machine learning; it is a core challenge in migration forecasting more broadly. Nonetheless, the approach offers a more flexible and scalable tool for near-term planning, particularly when used alongside scenario-based analysis that explicitly considers policy changes.

Substantively, our findings suggest that the UK’s international student landscape is undergoing a profound transformation. European Union student demand has collapsed following Brexit and shows little sign of recovery in the medium term. Applications from Mainland China and Hong Kong, long the largest source, appear to have peaked and are expected to decline gradually. India, a key growth market, is projected to plateau amid tightening migration policies and domestic volatility. Yet, the relative importance of Mainland China and Hong Kong, India and the European Union in the UK’s international student base is forecast to increase. This produces a concentration paradox in which reliance on a small set of origins intensifies even as their absolute contributions stagnate or fall, increasing systemic vulnerability.

These findings have direct implications for UK higher education and migration governance. International students have long been treated as a stable revenue stream within institutional and governmental planning, but our results suggest that this assumption is increasingly untenable. Policy changes—including restrictions on dependants^43^, tightening of post-study work pathways^44^, and proposals for an international student levy^45^—may achieve short-term political objectives around net migration but risk undermining the financial foundations of the sector. The Office for Students has warned that around 40% of English universities face deficits post-2024, partly driven by declining international student numbers^33,40^. This pressure is reflected in widespread redundancies and programme closures across the sector^46^. At the same time, the UK Home Office has placed greater onus on universities to recruit ‘genuine’ students, with several institutions temporarily pausing applications from Pakistan and Bangladesh^47^. These forecasts are also situated within the context of the publication of the UK’s recent International Education Strategy^48^, which emphasises sustainable recruitment and global competitiveness without imposing specific number targets for onshore student numbers, prioritising growth in education exports to £40 billion per year through a move towards transnational education and recruitment of ‘high-quality’ students. Forecasts such as those presented here can help quantify the longer-term implications of these policy trajectories and contextualise short-term declines within a system of growing dependence.

The patterns we document are not unique to the UK. International higher education systems globally are increasingly exposed to geopolitical instability and shifting migration regimes. In the United States, deteriorating relations with China and a more hostile migration policy environment during the first Trump administration led to declines in student numbers^49,50^. During the COVID-19 pandemic, anti-immigrant sentiment and discrimination against Asian populations were associated with falls in Chinese student enrolments in key destinations^51^. These examples illustrate how student mobility can serve as a barometer of broader political dynamics and how higher education is embedded within, rather than insulated from, migration politics.

From a migration-systems perspective, the concentration paradox observed in UK ISM raises questions about system resilience. If a small number of corridors underpin the financial viability of HEIs, shocks affecting those corridors can propagate rapidly through the system. Brexit’s impact on European Union student flows offers a cautionary example: a long-established migration corridor effectively collapsed within a few application cycles, reshaping the composition of international cohorts and leaving institutions scrambling to diversify recruitment.

Our forecasts suggest that, in the absence of deliberate diversification efforts or policy changes, the UK is on a path towards similar vulnerabilities with respect to Mainland China and Hong Kong and India. This does not imply that these relationships will necessarily deteriorate, but it highlights the strategic risk of over-reliance. Building resilience may require a mix of actions: diversifying origin markets; rethinking funding models to reduce dependence on international fees; and designing migration policies that balance political constraints with the need for stable, predictable student inflows.

The study has several limitations. First, while the XGBoost model offers improved predictive accuracy, its interpretability remains more limited than that of traditional regression models. Variable importance measures and partial dependence plots can offer insight into drivers, but the model is primarily used here as a forecasting tool rather than a causal inference framework. Institutions and policymakers who prioritise transparency may still prefer simpler models for certain applications.

Second, the evaluation period includes Brexit and COVID-19, making it inherently challenging to predict. The model’s performance in calmer periods might differ, and its accuracy under future shocks cannot be guaranteed. Third, important covariates such as exchange rates, detailed visa policies, diaspora community size and institutional prestige are not included due to data availability, particularly for the forecast horizon. Incorporating such variables could improve accuracy but would require consistent, prospective data.

Finally, we focus on undergraduate applications captured via UCAS. Our analysis relies on UCAS accepted applications rather than realised enrolments. While UCAS captures the majority of international undergraduate entrants and provides the most consistent and temporally granular administrative measure available across origin countries and time, conversion rates from acceptance to enrolment may vary across countries, subjects and contexts. Forecasts should therefore be interpreted as projections of international student demand and confirmed undergraduate placements, rather than guaranteed realised enrolments. At the same time, supplementary comparisons with publicly available HESA undergraduate data suggest that UCAS acceptances provide a reasonable proxy for both aggregate temporal dynamics and the composition of the principal origin countries analysed here. The weaker UCAS–HESA correspondence for Hong Kong may partly reflect differences between UCAS applicant domicile and HESA non-UK permanent-address reporting, particularly following the introduction of the British National Overseas route. In addition, UCAS data cover only undergraduate admissions and do not include all pathways into UK higher education, particularly at postgraduate level. Postgraduate taught and research students are an important part of the UK higher-education landscape and may follow different trajectories, and changes in their numbers may have distinct institutional and financial implications.

Future research could address these limitations in several ways. Extending the analysis to postgraduate and subject-specific applications would provide a more complete picture of risks and opportunities in the higher-education sector. Incorporating more detailed policy and economic indicators—including exchange rates, targeted scholarship schemes and visa-rule changes—could help to disentangle the drivers of observed shifts and refine forecasts. Methodologically, exploring alternative machine-learning architectures such as recurrent neural networks or attention-based models may offer further gains in accuracy, though with additional complexity. Finally, applying similar methods to other major destinations and to multi-destination systems would facilitate comparative analysis of global ISM patterns and shed light on how shocks and policies ripple through the broader migration system.

Ultimately, ISM remains central to the UK’s higher education landscape and its global position in knowledge production. In an era of heightened uncertainty, proactive diversification of origin markets, stable and transparent migration pathways, and investment in inclusive support for diverse student populations will be key to sustaining the benefits of ISM while mitigating the risks of an increasingly concentrated and politically contested system.

## Methods

### Data and preprocessing

This is a quantitative observational study based on secondary analysis of aggregated country-year administrative data. We use administrative data from the Universities and Colleges Admissions Service (UCAS), the centralised platform through which the majority of applicants apply for a full-time undergraduate degree at UK higher education institutions. UCAS captures approximately 95% of European Union undergraduate entrants and around 60% of non-European Union entrants^25^. We analyse accepted international undergraduate applications by country of domicile for the period 2010–2024, covering 86 countries of origin. These data were accessed through a formal research partnership with UCAS.

The dependent variable is the annual count of successful undergraduate applications from each origin country. In UCAS terminology, an acceptance refers to an applicant who has firmly accepted an offer of a place at a UK higher education institution through the UCAS admissions system. This stage follows the submission of an application and the receipt of one or more conditional or unconditional offers. An acceptance therefore represents a confirmed admissions placement and a high-commitment stage in the mobility decision process, although final enrolment remains contingent on meeting offer conditions and completing institutional registration.

UCAS acceptances provide a consistent, origin-disaggregated and temporally granular administrative series that is available with minimal delay. We therefore interpret them as a timely administrative proxy for undergraduate international student demand and mobility, while recognising that they represent admissions outcomes rather than realised enrolments or visa issuances. Comparisons with publicly available Higher Education Statistics Agency data indicate strong aggregate temporal alignment for European Union and non-European Union undergraduate entrants, close correspondence in the composition of the principal non-European Union origin countries highlighted in the analysis, and strong origin-specific temporal correspondence for most major sending countries. Additional sensitivity checks against Higher Education Statistics Agency all-student enrolment counts show similar temporal alignment for most major origins, while also confirming that broader enrolment-stock measures are less directly comparable to UCAS undergraduate admissions flows, particularly for Hong Kong (Supplementary Note 2).

To capture the drivers of these flows, we augment the UCAS data with origin- and destination-specific covariates drawn from the World Bank World Development Indicators, the CEPII Gravity Database, and the United Nations World Population Prospects. These include GDP per capita and population size in origin and destination countries, indicators for common official language and historical colonial ties, and great-circle distance between the origin country and the UK. The analytical panel includes all origin countries in the UCAS data with at least 10 successful applications over 2010–2024, resulting in 86 origin countries.

These covariates are selected based on prior evidence concerning the determinants of ISM^19,20,23^. For both the out-of-sample evaluation and the final forecast period, economic and demographic inputs are drawn from projection series available before the relevant prediction year. This ensures that predictions use information that would realistically have been available at the time of forecasting and avoids information leakage from subsequently realised values. For the final forecast horizon of 2025–2030, we use projection series released in 2024. The reliance on prospective covariates means that some known drivers of ISM, including institutional rankings and detailed measures of diaspora size, cannot be included because globally consistent projections are unavailable.

We construct a lagged application feature, *y*_*i*,*t* − 1_, representing the number of successful applications from origin country *i* in the previous year. This captures temporal persistence in application flows. Continuous covariates are standardised using z-scores to facilitate model convergence and comparability across features. As the outcome is a right-skewed count variable, we use modelling approaches appropriate for count data.

### Forecasting models

Our primary forecasting model is Extreme Gradient Boosting (XGBoost), a gradient-boosted decision-tree algorithm adapted for count data using a Poisson objective function. XGBoost incrementally fits an ensemble of regression trees, with each successive tree trained to improve predictions from the preceding ensemble^52^. This makes it well suited to capturing complex, non-linear relationships and interactions among predictors.

Let *y*_*i*_ denote the number of successful applications for observation *i*, representing a country-year pair, and let *x*_*i*_ denote the corresponding vector of covariates, including lagged applications, economic and demographic indicators, structural characteristics, and calendar year. XGBoost approximates the log of the expected count as shown in Eq. 1:

1 $$\log {\hat{y}}_{i}=f({x}_{i})={\sum}_{k=1}^{K}{f}_{k}({x}_{i}),$$

where each *f*_*k*_ is a regression tree and *K* is the number of boosting rounds. Under the Poisson objective, the loss function is defined in Eq. 2:

2 $$L(\theta )={\sum}_{i=1}^{n}\left[\exp \{f({x}_{i})\}-{y}_{i}f({x}_{i})\right]+\lambda {\sum}_{j}{\theta }_{j}^{2},$$

where *θ* denotes the set of tree parameters and *λ* is an L2 regularisation term controlling model complexity. Predicted application counts are obtained using Eq. 3:

3 $${\hat{y}}_{i}=\exp \{f({x}_{i})\}.$$

The XGBoost specification uses a learning rate of 0.3, a maximum tree depth of 6, and full row and column sampling. Five-fold cross-validation with early stopping over the training data is used to select the number of boosting rounds, which is typically approximately 150–200, as summarised in Supplementary Table 2. Further implementation details are provided in the Supplementary Methods.

To contextualise the added value of machine learning, we implement two benchmark forecasting approaches commonly used in sector planning and migration research.

First, we estimate autoregressive integrated moving average (ARIMA) models separately for each origin country. ARIMA models extrapolate historical trends in application counts without incorporating external covariates. Country-specific orders are selected using information-criterion-based optimisation. ARIMA therefore serves as a pragmatic time-series benchmark for assessing whether more flexible methods provide measurable gains in predictive performance.

Second, we estimate a negative binomial gravity-type model that incorporates time-varying economic and demographic characteristics and structural relationships including distance, common language and colonial ties. The model includes country-specific random intercepts to capture unobserved heterogeneity and a lagged dependent variable to represent temporal persistence. The gravity model provides a theoretically informed comparator to the machine-learning approach. Complete model equations and estimation details are presented in the Supplementary Methods.

### Model evaluation and forecasting strategy

For the principal evaluation, models are trained on data from 2010 to 2018 and evaluated over an out-of-sample period from 2019 to 2023. This period encompasses the implementation of Brexit and the COVID-19 pandemic, providing a stringent assessment of forecasting performance under structural volatility rather than smooth trend continuation. The time-based split mimics real-world forecasting and prevents realised post-2018 outcomes from entering the training data.

As an additional holdout exercise, the XGBoost model is trained on observations from 2010 to 2023 and used to predict 2024. The final forecasting model is then trained using observations through 2024 and used to generate forecasts for 2025–2030.

We evaluate predictive accuracy using standard forecast-error metrics. Mean absolute error is defined in Eq. 4:

4 $${{{{\rm{MAE}}}}}=\frac{1}{n}{\sum}_{i=1}^{n}\left\vert {y}_{i}-{\hat{y}}_{i}\right\vert,$$

and measures the average magnitude of prediction errors. Root mean squared error is defined in Eq. 5:

5 $${{{{\rm{RMSE}}}}}=\sqrt{\frac{1}{n}{\sum}_{i=1}^{n}{\left({y}_{i}-{\hat{y}}_{i}\right)}^{2}},$$

and assigns greater weight to larger errors. Normalised mean absolute error is defined in Eq. 6:

6 $${{{{\rm{NMAE}}}}}=\frac{{{{{\rm{MAE}}}}}}{\bar{y}},$$

where $\bar{y}$ is the mean number of applications for a given country. Normalisation enables comparison of relative forecasting error across countries with different application volumes. We also calculate mean absolute percentage error and the Pearson correlation between observed and predicted application counts. Definitions and implementation details for all evaluation metrics are provided in the Supplementary Methods under 'Evaluation metrics'.

After model evaluation, we retrain the XGBoost model on the full observed period from 2010 to 2024 and generate forecasts for 2025–2030 recursively. The forecast for 2025 uses observed lagged applications from 2024, while forecasts for 2026–2030 use model-predicted values for the lagged dependent variable. Economic and demographic covariates for 2025–2030 are taken from the relevant projected World Bank and United Nations World Population Prospects series.

To construct prediction intervals, we use a model-retraining bootstrap. Historical country-year observations are resampled with replacement, the XGBoost model is re-estimated using the selected hyperparameters, and recursive forecasts are generated for 2025–2030. This process is repeated 250 times. The 2.5th and 97.5th percentiles of the resulting forecast distributions form the bounds of the 95% prediction intervals around each point forecast.

ARIMA models are fitted separately for each origin country. The XGBoost and negative binomial gravity models are fitted to the country-year panel, with the gravity model including country-specific random intercepts. All models generate predictions at the origin-country level. Regional aggregates, including Mainland China and Hong Kong combined and the European Union, are constructed after prediction by summing origin-specific forecasts. Uncertainty intervals for aggregated series are obtained by summing bootstrap predictions across origins within each iteration and then computing empirical quantiles.

### Reporting summary

Further information on research design is available in the Nature Portfolio Reporting Summary linked to this article.

## Supplementary information

Supplementary Information

Reporting Summary

Transparent Peer Review File

## Data Availability

The UCAS admissions data used in this study were provided under licence by the Universities and Colleges Admissions Service and are not publicly available. Access to the UCAS data is restricted by UCAS licensing terms and can be requested through the authors, who will liaise with UCAS regarding permission for reuse. Public covariates used in this study are available from the World Bank World Development Indicators, the United Nations World Population Prospects and the CEPII Gravity Database.

## Code availability

The code used to process the data, estimate the forecasting models and generate all reported results is publicly available at https://github.com/ruthneville/ism_uk_forecasts. The repository includes scripts for data preprocessing, model estimation, forecasting and uncertainty quantification. All analyses were conducted in R version 4.5.0 (2025-04-11 ucrt) using xgboost 1.7.11.1, forecast 8.24.0, glmmTMB 1.1.13, dplyr 1.1.4, ggplot2 3.5.2 and tidyr 1.3.1.

## References

[1] UNESCO Institute for Statistics (UIS). Global flow of tertiary students, accessed 09 December 2025. http://uis.unesco.org/en/uis-student-flow. UNESCO Institute for Statistics (UIS).

[2] Saxenian, A. L. From brain drain to brain circulation: transnational communities and regional upgrading in India and China. *Stud. Comp. Int. Dev.* **40**, 35–61, 10.1007/BF02686293 (2005).

[3] Knight, J. *Student Mobility and Internationalisation: Trends and Policies* (UNESCO, 2012).

[4] Jon, J.-E. Realizing internationalization at home: Korean higher education institutions’ responses to internationalization. *J. Stud. Int. Educ.* **17**, 460–475, 10.1177/1028315312468329 (2013).

[5] Docquier, F. & Rapoport, H. Globalization, brain drain, and development. *J. Econ. Lit.* **50**, 681–730, 10.1257/jel.50.3.681 (2012).

[6] Robertson, S. Transnational student-migrants and the state: the education-migration nexus. *Popul. Space Place* **19**, 180–193, 10.1002/psp.1745 (2013).

[7] Czaika, M. & Parsons, C. R. The gravity of high-skilled migration policies. *J. Econ. Geogr.* **17**, 371–403, 10.1093/jeg/lbw041 (2017). 28290123

[8] Altbach, P. G. & Knight, J. The internationalization of higher education: motivations and realities. *J. Stud. Int. Educ.* **11**, 290–305, 10.1177/1028315307303542 (2007).

[9] Lomer, S. Uk policy discourses and international student mobility: the deterrence and subjectification of international students. *Glob. Soc. Educ.* **16**, 308–323, 10.1080/14767724.2017.1401454 (2017).

[10] She, Q. & Wotherspoon, T. International student mobility and high-skilled migration: a comparative study of Canada, the United States, and the United Kingdom. *SpringerPlus* **2**, 132. 10.1186/2193-1801-2-132 (2013). 23667802 PMC3648681

[11] Migration Observatory. Student migration to the UK, accessed 09 December 2025. https://migrationobservatory.ox.ac.uk/resources/briefings/student-migration-to-the-uk/ (2025).

[12] OECD. *Education at a Glance 2023*. 10.1787/69096873-en (OECD Publishing, 2023).

[13] Cuibus, M. & Walsh, P. *Student Migration to the UK* (Migration Observatory, 2024).

[14] Spivak, G. C. Rethinking comparativism. *New Lit. Hist.* **40**, 609–626 (2009).

[15] King, R. & Sondhi, G. International student migration: a comparison of UK and Indian students’ motivations for studying abroad. *Glob. Soc. Educ.* **16**, 176–191 (2018).

[16] Wei, H. An empirical study on the determinants of international student mobility: a global perspective. *High. Educ.* **66**, 105–122 (2013).

[17] Caruso, R. & De Wit, H. Determinants of mobility of students in Europe: empirical evidence for the period, 1998-2009. *J. Stud. Int. Educ.* **19**, 265–282, 10.1177/1028315314563079 (2014).

[18] Massey, D. S. et al. Theories of international migration: a review and appraisal. *Popul. Dev. Rev.* **19**, 431–466 (1993).

[19] Beine, M., Noël, R. & Ragot, L. Determinants of the international mobility of students. *Econ. Educ. Rev.* **41**, 40–54 (2014).

[20] Neville, R., Rowe, F. & Singleton, A. Nonlinear relationships between human development and international student mobility: the prominent role of employment prospects and cultural and linguistic ties. *Popul. Space Place* **30**, e2610, 10.1002/psp.2610 (2024).

[21] Lewer, J. J. & Van den Berg, H. A gravity model of immigration. *Econ. Lett.* **99**, 164–167 (2008).

[22] de Haas, H. Migration and development: a theoretical perspective. *Int. Migr. Rev.* **44**, 227–264, 10.1111/j.1747-7379.2009.00804.x (2010). 26900199 PMC4744987

[23] Weber, T. & van Mol, C. The student migration transition: an empirical investigation into the nexus between development and international student migration. *Comp. Migr. Stud.* **11**. 10.1186/s40878-023-00351-z (2023). PMC10068235 37033417

[24] Bolton, P., Lewis, J. & Gower, M. International Students in UK Higher Education. Technical Report CBP 7976, accessed 09 December 2025. https://dera.ioe.ac.uk/id/eprint/40450/1/CBP-7976.pdf. (House of Commons Library, 2023).

[25] UCAS. 2019 International Insights Report. Accessed 18 July 2025. https://www.ucas.com/data-and-analysis/ucas-international-insights. (2019).

[26] Bolton, P. & Lewis, J. The End of Free Movement and the Implications for Higher Education. Technical Report CBP 9187, accessed 09 December 2025. https://researchbriefings.files.parliament.uk/documents/CBP-9187/CBP-9187.pdf. (House of Commons Library, 2021).

[27] Neville, R., Rowe, F. & Singleton, A. Unraveling the Brexit–COVID-19 nexus: assessing the decline of EU student applications into UK universities. *Data Policy* **6**, e45, 10.1017/dap.2024.45 (2024).

[28] Amuedo-Dorantes, C. & Romiti, A. International student applications in the UK after Brexit. *J. Econ. Geogr.* **24**, 637–662, 10.1093/jeg/lbae019 (2024).

[29] Department of Education and Department for International Trade. International education strategy: global potential, global growth. https://www.gov.uk/government/publications/international-education-strategy-global-potential-global-growth (2021).

[30] UK Home Office. Immigration Rules: Changes to Appendix Student and Appendix Graduate. https://www.gov.uk/government/publications/statement-of-changes-immigration-rules-hc-1160-17-july-2023. (Policy Statement, 2023).

[31] UK Home Office. New package of measures to cut net migration and tackle abuse of the student route, accessed 09 December 2025. https://www.gov.uk/government/news/new-package-of-measures-to-cut-net-migration-and-tackle-abuse-of-the-student-route. (2024).

[32] Universities UK. *The Impact of Reforms to the Graduate Route and Dependant Rules*. Technical Report (Universities UK, 2024).

[33] Lapworth, S. Universities fighting for their future must be bold and must be fast. Accessed 18 July 2025. https://www.officeforstudents.org.uk/news-blog-and-events/blog/universities-fighting-for-their-future-must-be-bold-and-must-be-fast/. (Office for Students Blog, 2024).

[34] National Research Council. *Beyond Six Billion: Forecasting the World’s Population* (National Academy Press, 2000).

[35] Disney, G., Wiśniowski, A., Forster, J. J., Smith, P. W. F. & Bijak, J. *Evaluation of Existing Migration Forecasting Methods and Models: Report Examining and Assessing the Various Analytical Approaches to Migration Forecasting*. Technical Report. https://www.gov.uk/government/publications/evaluation-of-existing-migration-forecasting-methods-and-models (ESRC Centre for Population Change, University of Southampton, 2015).

[36] British Council. The Outlook for International Student Mobility: Amidst a Changing Global Macroeconomic Landscape (British Council, 2024).

[37] Bijak, J., Hilton, J., Silverman, B. W. & Smith, P. W. F. Assessing time series models for forecasting international migration: lessons from the United Kingdom. *J. Forecast.* **38**, 470–487 (2019).

[38] Azose, J. J. & Raftery, A. E. Bayesian probabilistic projection of international migration. *Demography* **52**, 1627–1650 (2015). 10.1007/s13524-015-0415-0 PMC4605963 26358699

[39] Cabanas-Tirapu, O., Danús, L., Moro, E., Sales-Pardo, M. & Guimerà, R. Human mobility is well described by closed-form gravity-like models learned automatically from data. *Nat. Commun.* **16**, 1336. 10.1038/s41467-025-56495-5 (2025). 39904994 PMC11794706

[40] Office for Students. *Financial Sustainability of Higher Education Providers in England: 2024*. Technical Report, accessed 09 December 2025, https://www.officeforstudents.org.uk/publications/financial-sustainability-of-higher-education-providers-in-england-2024/. (Office for Students, 2024).

[41] Litmeyer, M.-L. & Hennemann, S. Forecasting first-year student mobility using explainable machine learning techniques. *Rev. Reg. Res.* **44**, 119–140, 10.1007/s10037-024-00207-x (2024).

[42] Yang, S.-W., Chen, H.-C., Chen, W.-C. & Yang, C.-H. Forecasting outbound student mobility: a machine learning approach. *PLoS ONE* **15**, e0238129 (2020). 10.1371/journal.pone.0238129 PMC7470415 32881900

[43] UK Home Office. Statement of changes to the immigration rules: HC 1160, accessed 09 December 2025. https://www.gov.uk/government/publications/statement-of-changes-immigration-rules-hc-1160-17-july-2023. (2023).

[44] Migration Advisory Committee. *Rapid Review of the Graduate Route*. Technical Report, https://www.gov.uk/government/publications/rapid-review-of-the-graduate-route-may-2024 (Migration Advisory Committee, 2024).

[45] Centre for Policy Studies. *Stopping the Brain Drain: A Student Visa Levy for the UK*. Technical Report, https://cps.org.uk/research/stopping-the-brain-drain/, (Centre for Policy Studies, 2023).

[46] Adams, R. More than 40 UK universities planning job cuts amid financial strain. *The Guardian*. https://www.theguardian.com/education/2024/mar/05/uk-universities-job-cuts-financial-crisis (2024).

[47] Financial Times. UK universities restrict recruitment of Bangladeshi and Pakistani students over visa concerns. *Financial Times*, accessed 17 July 2026. https://www.ft.com/content/7b98c0b2-7327-495b-815e-65af5a172499. (2025).

[48] Department for Education and Department for Business and Trade. The UK International Education Strategy 2026. https://assets.publishing.service.gov.uk/media/696a6164448fedc1eb4248ef/international-education-strategy-2026.pdf. (UK Government Policy Document, 2026).

[49] Rose-Redwood, C.-A. & Rose-Redwood, R. Rethinking the politics of the international student experience in the age of Trump. *J. Int. Stud.* **7**, I–X, 10.32674/jis.v7i3.201 (2017).

[50] Laws, K. N. & Ammigan, R. International students in the Trump era: a narrative view. *J. Int. Stud.* **10**, xviii–xxii, 10.32674/jis.v10i3.2841 (2020).

[51] Rowe, F., Mahony, M., Graells-Garrido, E., Rango, M. & Sievers, N. Using Twitter to track immigration sentiment during early stages of the COVID-19 pandemic. *Data Policy* **3**, e9. 10.1017/dap.2021.9 (2021).

[52] Migration Data Portal. International students. Global Migration Data Portal, International Organization for Migration (IOM). https://www.migrationdataportal.org/themes/international-students-trends (accessed 1 September 2026).

