# Supplementary material for: Machine learning forecasts suggest a concentration paradox in international student mobility to the United Kingdom
Source: Nat Commun. 2026 Sep 17;17:9604. doi: 10.1038/s41467-026-77425-z (PMC13586154; doi:10.1038/s41467-026-77425-z)
Supplement: Supplementary file 1 [file 41467_2026_77425_MOESM1_ESM.pdf]

Supplementary Information

Machine Learning Forecasts Suggest a Concentration Paradox in International Student Mobility to the United Kingdom

Ruth Neville[1], Francisco Rowe[2], Emilio Zagheni[3]

[1] Centre for Advanced Spatial Analysis (CASA), University College London, London, UK
[2] Department of Geography and Planning, University of Liverpool, Liverpool, UK
[3] Max Planck Institute for Demographic Research, Rostock, Germany

[*] Corresponding author: ruth.neville@ucl.ac.uk

Contents

1. Supplementary Methods
2. Supplementary Note 1 — Extended performance results
3. Supplementary Note 2 — Validation of UCAS acceptances against public HESA data
4. Supplementary Note 3 — Reproducibility and code availability
5. Supplementary References

Supplementary Methods

Data sources, preprocessing, and coverage

This study draws on administrative data from the Universities and Colleges Admissions Service (UCAS), which provides the principal route through which students apply for undergraduate degrees at UK higher education institutions. UCAS data capture approximately 95% of entrants from the European Union (EU) and around 60% from non-EU countries, making them a robust representation of undergraduate international student mobility to the UK.

We focus on successful international undergraduate applications (acceptances) by country of domicile for the period 2010–2024. The dependent variable is the annual count of successful acceptances from each origin country.

To capture drivers of these flows, we augment the UCAS data with origin- and destination-specific covariates widely used in the migration and international student mobility literature. These include economic and demographic indicators (GDP per capita and population size), structural relationships (common official language, historical colonial ties), and bilateral distance (great-circle distance to the UK). Covariates are obtained from the World Bank World Development Indicators, the United Nations World Population Prospects (WPP, 2022 Revision), and the CEPII Gravity Database.

Projected covariates and the information set at prediction time

For both the forecast period (2025–2030) and the evaluation period (2019–2023), we rely on externally produced projections rather than realised values for economic and demographic covariates. This ensures that forward-looking predictions are based on information that would have been available at the time of forecasting, thereby avoiding information leakage.

Specifically, projections for GDP per capita and population are taken from the most recent World Bank and WPP releases available prior to the forecast year. For example, predictions for 2019 use projection series released before 2019; predictions for 2024 use series released in 2019. For the final forecast horizon (2025–2030) we use projections released in 2024.

This design mirrors realistic decision-making conditions, where policy makers must rely on macroeconomic and demographic forecasts rather than their eventual realisation. It also necessitates the omission of some known drivers of international student mobility—such as institution-specific rankings, subject-level composition, and detailed measures of diaspora size—for which no globally consistent prospective data are available.

Preprocessing and feature engineering

Several preprocessing steps and feature engineering operations are applied prior to model estimation:

- **Standardisation of covariates.** Continuous covariates (GDP per capita and population size for origin and destination, and distance) are standardised to have mean zero and unit variance. This facilitates model convergence and comparability of feature scales, particularly for the XGBoost model.

- **Lagged dependent variable.** We construct a lagged application feature, $y_{i,t-1}$, representing the number of successful applications from origin country i in the previous year. This captures temporal persistence in application flows.

- **Recursive extension to 2030.** For the forecasting period (2025–2030), the model uses projected covariates and, for the lagged response, its own predicted values. Forecasts for year t thus depend on forecasts from year $t-1$, reflecting the recursive nature of the forecasting process.

- **Country inclusion criteria.** To reduce the influence of highly stochastic series, we exclude origin countries with fewer than 10 total successful applications over the full 2010–2024 period. The final dataset comprises 86 origin countries.

A summary of variables, definitions, temporal coverage, and data sources is presented in Supplementary Table 1.

Supplementary Table 1 — Covariates, definitions, and data sources.

**Variable**	**Definition**	**Source**	**Temporal coverage**
Applications	Annual count of successful undergraduate acceptances from origin country to the UK	UCAS	2010–2024
GDP per capita (origin)	GDP per capita (constant prices), origin country	World Bank	2010–2030 (observed + projected)
GDP per capita (destination)	GDP per capita, UK	World Bank	2010–2030 (observed + projected)
Population (origin)	Total population, origin country	UN WPP	2010–2030 (observed + projected)
Population (destination)	Total population, UK	UN WPP	2010–2030 (observed + projected)
Distance	Great-circle distance between capital city of origin and London	CEPII	Time-invariant
Common language	Indicator for shared official language (English) between origin and UK	CEPII	Time-invariant
Colonial tie	Indicator for historical colonial link with the UK	CEPII	Time-invariant
Lagged applications	Successful applications from origin country in the previous year	Constructed	2011–2030
Year	Calendar year	Constructed	2010–2030

Forecasting models

We compare three forecasting approaches: a time-series benchmark (ARIMA), a theory-aligned regression model (negative binomial gravity), and a machine learning model (XGBoost). XGBoost is the primary model used to generate forward-looking forecasts.

ARIMA benchmark. We estimate autoregressive integrated moving average (ARIMA) models separately for each origin country. ARIMA models extrapolate historical trends in application counts without incorporating external covariates and are widely used in institutional planning and migration forecasting.

Let y_t denote the annual number of successful applications from a given origin country. The ARIMA(p, d, q) model can be written as:

$$\hat{y}_t = \mu + \sum_{i=1}^{p} \phi_i y_{t-i} - \sum_{j=1}^{q} \theta_j e_{t-j} + e_t, \tag{1}$$

where μ is a constant term, ϕ_i are autoregressive (AR) coefficients, θ_j are moving average (MA) coefficients, and e_t are residual errors. Differencing of order d is applied as required to achieve stationarity.

Country-specific orders (p, d, q) are selected using information-criterion–based optimisation (AICc). We use the `auto.arima()` function from the `forecast` package in R to identify the best-fitting model for each origin. Across origin countries, selected specifications predominantly feature first-order differencing and low-order AR and/or MA components, consistent with relatively short annual series. As ARIMA serves as a pragmatic time-series benchmark rather than a structural model of international student mobility, we do not tabulate all country-specific orders for brevity.

Negative binomial gravity model. To provide a theory-aligned comparator that incorporates structural, economic, and demographic characteristics, we specify a gravity-inspired negative binomial model for count outcomes.

Let y_{it} denote the number of successful applications from origin country i in year t. We model the conditional mean as:

$$\mathbb{E}(y_{it} \mid \cdot) = \lambda_{it} = \exp\left(\alpha + \alpha_i + \beta_1 d_i + \beta_2 s_i + \beta_3 p_{it} + \beta_4 p_{jt} + \rho y_{i,t-1}\right), \tag{2}$$

where:

- α is a global intercept;
- α_i is a country-specific random intercept capturing unobserved heterogeneity;
- d_i is the log of great-circle distance between origin i and the UK;
- s_i is a vector of structural indicators (common language, colonial tie);
- p_{it} is a vector of time-varying origin-country characteristics (e.g., GDP per capita, population);

- p_{jt} is a vector of time-varying destination-country characteristics (e.g., UK GDP per capita, population);
- $y_{i,t-1}$ is the lagged dependent variable capturing temporal dependence, with coefficient ρ.

We assume a negative binomial distribution for y_{it} with mean λ_{it} and overdispersion parameter κ, allowing variance to exceed the mean, as is typical in migration and application count data. The model is estimated using the `glmmTMB` package in R, with a log link and random intercepts at the country level.

Parameter estimates are directionally consistent with prior work on migration and international student mobility: GDP per capita and population sizes are positively associated with application volumes, distance is negatively associated, and the lagged dependent variable captures strong temporal persistence. As the gravity model is included as a benchmark rather than for substantive interpretation of individual coefficients, we summarise these patterns narratively and do not reproduce the full coefficient table here.

XGBoost. Our primary forecasting model is Extreme Gradient Boosting (XGBoost), implemented with a Poisson objective to reflect the count nature of the outcome and the log-link structure used in the gravity model.

Let y_i denote the number of successful applications for observation i (a country–year pair), and let x_i be the vector of covariates including lagged applications, economic indicators, demographic variables, structural characteristics, and the calendar year. XGBoost approximates the log of the expected count via an additive ensemble of regression trees:

$$\log \hat{y}_i = f(x_i) = \sum_{k=1}^{K} f_k(x_i), \tag{3}$$

where each f_k is a decision tree and K is the number of boosting rounds. Under the Poisson objective, the loss function is:

$$L(\theta) = \sum_{i=1}^{n} [\exp\{f(x_i)\} - y_i f(x_i)] + \lambda \sum_{j} \theta_j^2, \tag{4}$$

where θ denotes the set of tree parameters and λ is an L2 regularisation term controlling model complexity. Predicted application counts are obtained as:

$$\hat{y}_i = \exp\{f(x_i)\}. \tag{5}$$

Hyperparameter tuning. Hyperparameters are selected using five-fold cross-validation with early stopping on the training period. We train candidate models on data from 2010–2018 and evaluate performance on 2019–2023, varying the number of boosting rounds and allowing the cross-validation procedure to select the optimal number via early stopping. In practice, we maintain a maximum depth of 6 and learning rate of 0.3, with the number of boosting rounds typically around 150–200, trading off flexibility against overfitting.

The main hyperparameter configuration is summarised in Supplementary Table 2. This specification is then used for the additional holdout exercise (training 2010–2023, predicting

2024) and for the final forecasting model (training 2010–2024, forecasting 2025–2030).

Supplementary Table 2 — XGBoost hyperparameter configuration.

Hyperparameter	Value	Notes
Objective	`count:poisson`	Poisson regression for modelling count outcomes
Max depth	6	Controls the maximum depth of each tree
Learning rate (`eta`)	0.3	Shrinks the contribution of each tree
Number of boosting rounds	∼150–200	Selected via five-fold cross-validation with early stopping
Subsample	1.0	No row subsampling used
Column subsampling	1.0	No feature subsampling used
L2 regularisation (`lambda`)	Via early stopping	Helps reduce overfitting

Forecasting strategy, evaluation, and uncertainty

Train-test splits and recursive forecasting. For the principal evaluation, models are trained on 2010–2018 and evaluated on 2019–2023. As an additional holdout exercise, the XGBoost model is trained on 2010–2023 and used to predict 2024. The final model is then trained using observations through 2024 and used to generate recursive forecasts for 2025–2030.

This period encompasses the implementation of Brexit and the COVID-19 pandemic, providing a stringent test of model performance under structural volatility rather than smooth trend continuation. Forecasts are produced recursively: the forecast for 2025 uses observed lagged applications from 2024, while forecasts for 2026–2030 use model-predicted values for the lagged dependent variable.

Evaluation metrics. We evaluate predictive accuracy using four standard measures of forecast error and one measure of association between predicted and realised values.

Mean absolute error (MAE).

$$\mathrm{MAE} = \frac{1}{n}\sum_{i=1}^{n} \left| y_i - \hat{y}_i \right|. \tag{6}$$

Root mean squared error (RMSE).

$$\mathrm{RMSE} = \sqrt{\frac{1}{n}\sum_{i=1}^{n} (y_i - \hat{y}_i)^2}. \tag{7}$$

Normalised mean absolute error (NMAE).

$$\mathrm{NMAE} = \frac{\mathrm{MAE}}{\bar{y}}, \tag{8}$$

where $\bar{y}$ is the mean number of applications for a given country. NMAE enables relative comparison of predictive performance across countries with different application volumes.

Mean absolute percentage error (MAPE).

$$\mathrm{MAPE} = \frac{1}{n}\sum_{i=1}^{n} \left| \frac{y_i - \hat{y}_i}{y_i} \right| \times 100. \tag{9}$$

MAPE provides an intuitive percentage measure of prediction error but can be inflated for small denominators. For origin countries recording fewer than approximately 50 applications annually, MAPE values should therefore be interpreted with caution.

Pearson correlation. We compute the Pearson correlation coefficient between predicted and observed values to summarise the strength of linear association across the evaluation period.

Aggregate performance statistics pooled across origin countries and years 2019–2023 are summarised in Supplementary Table 3, with country-level out-of-sample predictions shown in Supplementary Figure 1.

Supplementary Table 3 — Aggregate performance of benchmark and primary models, 2019–2023.

Model	MAE	RMSE	MAPE (%)	Correlation
ARIMA	327	1065	88	0.87
Negative binomial gravity	282	818	77	0.93
XGBoost	255	713	81	0.94

Bootstrap prediction intervals. To quantify uncertainty around the XGBoost forecasts, we construct 95% prediction intervals using a model-retraining bootstrap. For each forecast year and origin country:

1. We draw a bootstrap sample of the historical training data by resampling country–year observations with replacement.
2. We re-estimate the XGBoost model on this bootstrap sample using the same hyperparameters.
3. We generate a prediction for the focal forecast year (and, in the recursive setting, update the lagged dependent variable with the bootstrap prediction).

This process is repeated 250 times, yielding a distribution of forecast values for each country–year. The 2.5th and 97.5th percentiles of this distribution form the bounds of the 95% prediction interval. Because forecasts for later years depend on earlier predicted values, interval widths naturally widen with the forecast horizon, reflecting increasing uncertainty and the accumulation of structural volatility over time.

Supplementary Note 1 — Extended performance results

Country-level out-of-sample predictions from the primary model (2019–2023)

Supplementary Figure 1 shows out-of-sample predictions from the XGBoost model for the 2019–2023 evaluation period. Coloured lines represent model-predicted counts, and black points represent observed values. The figure provides a country-level view of validation performance and highlights three characteristics of the forecasting environment:

1. **Scale effects:** alignment is closer in absolute terms for high-volume sending countries, where signal dominates stochastic variation, while proportional errors are comparatively larger for low-volume countries.

2. **Volatility and discontinuity:** temporary divergences align with periods of structural disruption, such as the COVID-19 pandemic and policy changes affecting visa or fee status.

3. **Heterogeneity in trend persistence:** some countries exhibit smooth trend continuation, whereas others display non-linear trajectories that are challenging for all forecasting approaches.

This figure complements the aggregate performance metrics presented in Section 3 of the main paper by illustrating how system characteristics—rather than model specification alone—shape predictive accuracy.

Separate origin-level forecasts for Mainland China and Hong Kong

Mainland China and Hong Kong are treated as distinct origin systems throughout model estimation and forecasting. Given substantive differences in policy exposure and mobility dynamics between Mainland China and Hong Kong, we present separate origin-level forecasts to demonstrate distinct trajectories and uncertainty ranges in Supplementary Figure 2 . Mainland China and Hong Kong are reported as separate domiciles in the UCAS data, and do experience different relationships between their student mobility to the UK. For example, students from Hong Kong do have a British National Overseas (BNO) visa arrangement, perhaps making it easier to study in the UK. Hong Kong's colonial history with the UK is also likely to lower barriers to entry in terms of language, administration, and culture - as explored in[1]. However, the substantive text refers to aggregated patterns of Mainland China and Hong Kong.

Aggregated values shown in the main figures (e.g., Mainland China and Hong Kong combined) are constructed after prediction by summing origin-specific forecasts. Uncertainty intervals are aggregated within bootstrap iterations by summing bootstrap draws across origins and then taking quantiles.

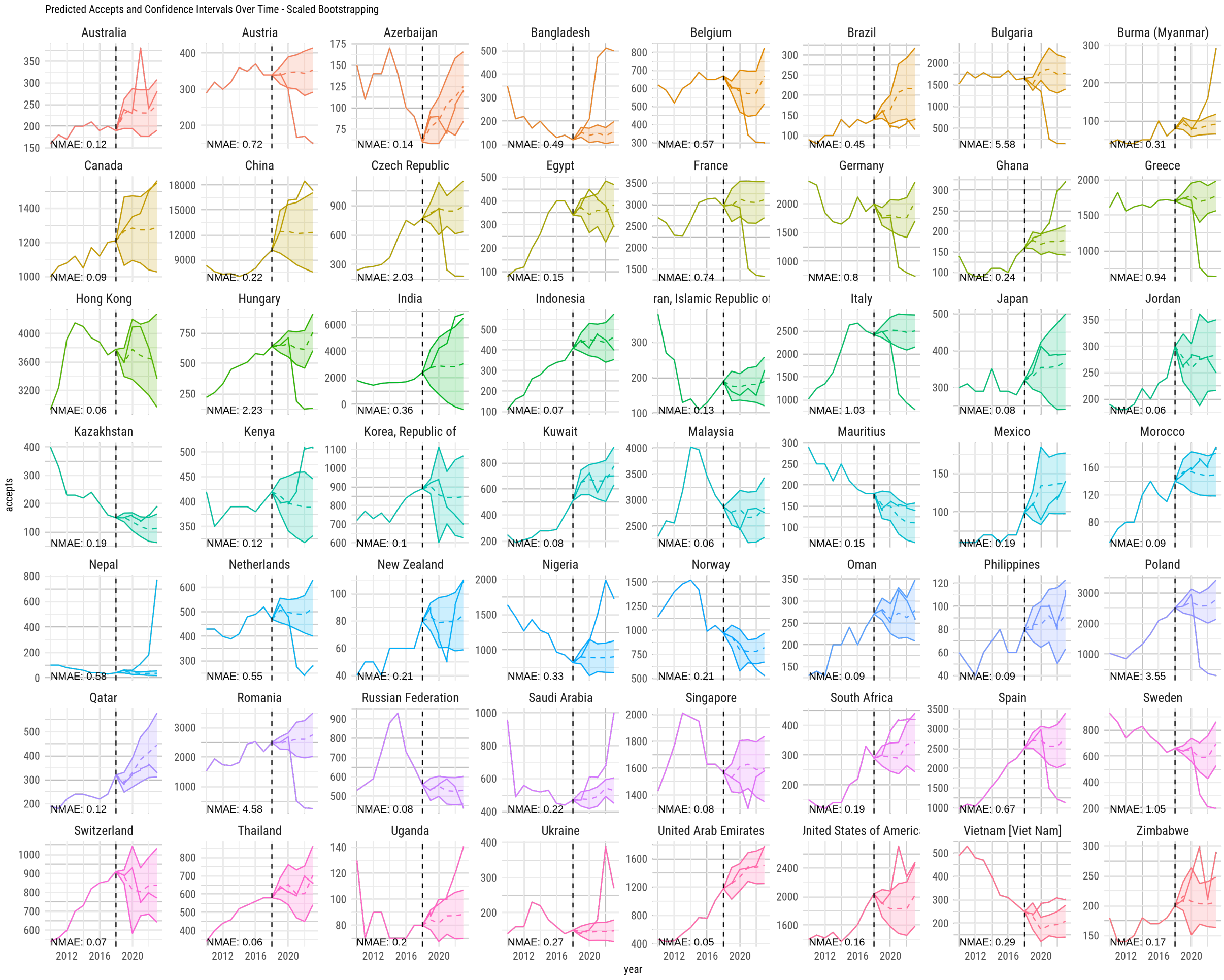

Supplementary Figure 1 — Country-level out-of-sample predictions from the XG-Boost model, 2019–2023. Panels display predicted (dashed lines) and observed (solid line) application counts for 60 major origin countries and NMAE for each country. The figure illustrates heterogeneous predictive accuracy shaped by country size, volatility, and exposure to structural shocks.

Supplementary Note 2 — Validation of UCAS acceptances against public HESA data

To assess whether UCAS undergraduate acceptances provide a reasonable proxy for broader international undergraduate mobility patterns, we compared the UCAS series used in the main analysis with publicly available HESA undergraduate statistics. These checks are intended as validation exercises rather than as evidence of equivalence between the two data sources. UCAS records acceptances within the admissions system, whereas HESA records undergraduate entrants or enrolments, and the two sources differ in timing, coverage, and administrative definition.

Aggregate entrant validation over time

For the main validation exercise, we used the public HESA Figure 9 extract filtered to entrant marker = Entrant, level of study = All undergraduate, mode of study = All, and country of HE

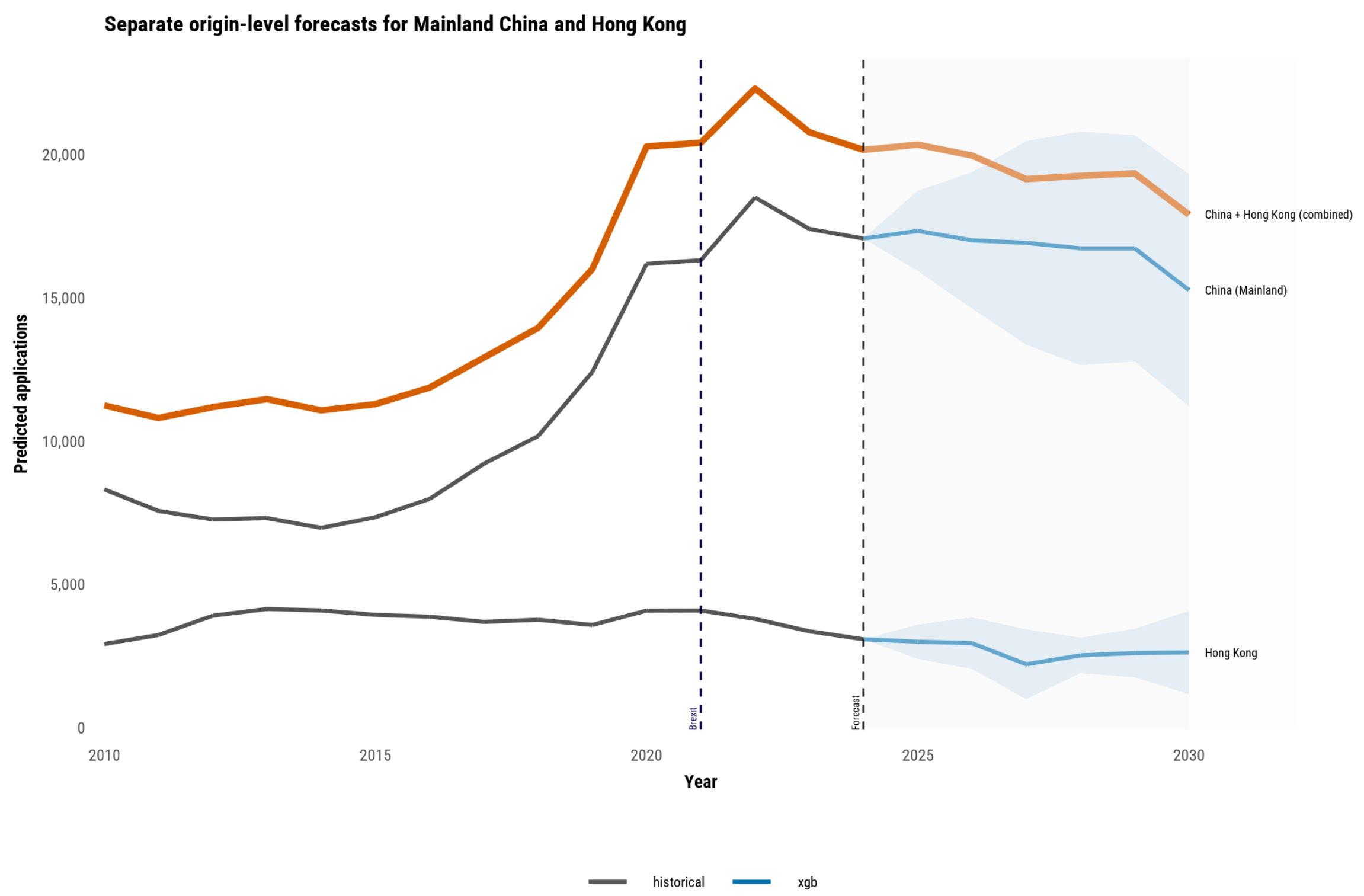

Supplementary Figure 2 — Separate origin-level forecasts for Mainland China and Hong Kong. Observed acceptances (2010–2024) are shown in grey; XGBoost forecasts for 2025–2030 are shown in blue with shaded 95% bootstrap prediction intervals. Predictions are generated separately for Mainland China and Hong Kong at the origin–year level. The orange line shows the combined China and Hong Kong series, constructed by aggregating origin-level forecasts, for comparison.

provider = All. Because the public HESA entrant table is available only at aggregate domicile-group level, we compared UCAS and HESA for two groups: European Union and non-European Union.

For each group, we aggregated the UCAS series to the corresponding category and calculated the Pearson correlation between annual UCAS acceptances (2020–2024) and HESA undergraduate entrant counts for the overlapping academic years (2020/21–2024/25). This correlation measures similarity in temporal movement over time rather than equality in absolute levels.

The resulting correlations are reported in Supplementary Table 4. Correspondence was very strong for both groups, indicating close temporal co-movement between UCAS acceptances and HESA undergraduate entrants at aggregate level for European Union students ($r = 0.998$) and Non-European Union students ($r = 0.975$).

Comparison with HESA undergraduate enrolment counts

To provide additional evidence beyond aggregate categories and a single-year composition check, we used the yearly public HESA country-of-domicile files: Table 28, "Non-UK domiciled HE students by HE provider and country of domicile, 2014/15 onward". These files were filtered to undergraduate study only (First degree and Other undergraduate), aggregated across providers,

Supplementary Table 4 — Aggregate and composition-based validation of UCAS against public HESA undergraduate data. Aggregate entrant comparisons use public HESA Figure 9 undergraduate entrant totals and should be interpreted as validation exercises rather than like-for-like equivalents of UCAS acceptances.

**Comparison**	**Years / units**	**Pearson** r
EU: UCAS acceptances vs HESA undergraduate entrants	2020–2024	0.998
Non-EU: UCAS acceptances vs HESA undergraduate entrants	2020–2024	0.975

and aligned to the corresponding UCAS annual acceptances series for the principal Non-EU origins highlighted in the paper.

We calculated Pearson correlations between the UCAS and HESA series over the overlapping period 2014–2024 for each origin separately. These results, reported in Supplementary Table 5, indicate strong temporal correspondence for most of the major Non-EU origin systems: China ($r = 0.973$), India ($r = 0.984$), Malaysia ($r = 0.860$), Nigeria ($r = 0.925$), Singapore ($r = 0.812$), the United Arab Emirates ($r = 0.989$), and the United States ($r = 0.886$). Correspondence was weaker for Hong Kong ($r = 0.670$), consistent with the fact that UCAS acceptances and HESA undergraduate enrolments are related but non-equivalent measures and may differ more for some origins than for others. These correlations indicate that UCAS and HESA series exhibit consistent co-movement over time across the principal origin countries, despite differences in absolute levels arising from coverage and definition.

We also examined the average gap between the two sources over time. Across all origins, HESA undergraduate enrolment counts are consistently higher than UCAS acceptances, reflecting differences in coverage and the fact that HESA captures all enrolled students (including non-UCAS routes and continuing cohorts), whereas UCAS captures admissions flows within a single application cycle. These gap statistics therefore help contextualise the level differences between the datasets, while the correlations summarise the extent to which they move together over time.

Supplementary Table 5 — Origin-specific temporal correspondence between UCAS acceptances and public HESA undergraduate enrolments, 2014–2024. Correlations are calculated between annual UCAS acceptances and yearly public HESA country-of-domicile undergraduate enrolment counts aggregated across providers for 2014–2024. HESA values are drawn from Table 28, "Non-UK domiciled HE students by HE provider and country of domicile, 2014/15 onward", and should be interpreted as a validation check rather than a direct equivalent of UCAS acceptances.

**Origin**	**Pearson** r
China	0.973
Hong Kong	0.670
India	0.984
Malaysia	0.860
Nigeria	0.925
Singapore	0.812
United Arab Emirates	0.989
United States	0.886

Comparison with HESA all-student enrolment counts

As an additional robustness check, we compared annual UCAS undergraduate acceptances with all-student enrolment counts from the same public HESA country-of-domicile table. This comparison uses records aggregated across all levels and modes of study, including undergraduate and postgraduate students, full-time and part-time modes, and both new and continuing enrolments. It is therefore broader than, and not directly equivalent to, the UCAS undergraduate acceptances series, which captures undergraduate admissions flows.

The results show strong temporal alignment for most major origins, although the correspondence is weaker than in the undergraduate-only validation checks for some comparisons. Correlations between UCAS undergraduate acceptances and HESA all-student enrolment counts were high for China ($r = 0.957$), India ($r = 0.991$), Malaysia ($r = 0.940$), Nigeria ($r = 0.951$), Singapore ($r = 0.807$), the United Arab Emirates ($r = 0.970$), and the United States of America ($r = 0.953$). Hong Kong was the main exception, with a weaker correlation ($r = 0.366$). The selected-origin composition correlation for 2024 was also lower for the all-student HESA comparison ($r = 0.787$) than for the undergraduate-only HESA comparison ($r = 0.978$), consistent with the broader and less directly comparable nature of the all-student HESA measure.

Supplementary Table 6 — UCAS–HESA validation using HESA all-student enrolment counts. Annual origin-level correlations compare UCAS undergraduate acceptances with all-student enrolment counts by country of permanent address from HESA Table 28 for 2014–2024. HESA all-student counts are aggregated across undergraduate and postgraduate levels, full-time and part-time modes, and both new and continuing enrolments, so these comparisons should be interpreted as sensitivity checks rather than direct equivalence tests.

**Origin**	**Pearson r**
China	0.957
Hong Kong	0.366
India	0.991
Malaysia	0.940
Nigeria	0.951
Singapore	0.807
United Arab Emirates	0.970
United States of America	0.953
**Selected-origin composition, 2024**	
UCAS vs HESA all students	0.787

Interpretation and limitations

Taken together, these checks support the use of UCAS acceptances as a reasonable administrative indicator of broad patterns in undergraduate international student mobility. Aggregate validation shows that UCAS tracks public HESA undergraduate entrant dynamics closely for EU and non-EU groups, while the country-composition and origin-specific analyses indicate similar correspondence for the major non-EU origins emphasised in the paper. The additional all-student HESA comparison further shows that broad temporal alignment remains strong for most major origins even when compared with wider enrolment-stock measures, although the

lower composition correlation confirms that all-student HESA counts are less directly comparable to UCAS undergraduate acceptances than undergraduate-specific HESA measures.

However, these checks do not remove the conceptual distinction between admissions outcomes and realised enrolments. UCAS acceptances should therefore be interpreted as confirmed undergraduate admissions placements within the UCAS system rather than exact measures of final enrolment.

Several limitations should be noted. First, UCAS records acceptances, whereas HESA records entrants or enrolments. Second, HESA public data are reported by academic year, whereas the UCAS analytical panel is organised by calendar year. Third, public HESA entrant data are available only at broad domicile-group level rather than as a full country-year panel. Fourth, the DT051 country tables are undergraduate-only but not entrant-specific.

Hong Kong is a clear exception in the origin-specific comparisons. One plausible explanation is that UCAS records applicant domicile at the admissions stage, whereas HESA's public country-of-domicile table reports students by non-UK permanent address. Following the introduction of the British National Overseas route, some Hong Kong-origin students may have reported, or later come to report, a UK permanent address by the time of enrolment or continued study, reducing their visibility in HESA's Hong Kong non-UK permanent-address counts.

Supplementary Note 3 — Reproducibility and code availability

All modelling was conducted in R version 4.5.0 using `xgboost` 1.7.11.1, `forecast` 8.24.0, `glmmTMB` 1.1.13, `dplyr` 1.1.4, `ggplot2` 3.5.2, and `tidyr` 1.3.1 on standard CPU-based computing resources. Code for data preprocessing, model estimation, evaluation, forecasting and figure generation is publicly available at `https://github.com/ruthneville/ism_uk_forecasts`.

Supplementary References

[1] Ruth Neville, Francisco Rowe, and Alex Singleton. Nonlinear relationships between human development and international student mobility: The prominent role of employment prospects and cultural and linguistic ties. *Population, Space and Place*, 30(5), 2024.
