# Supplementary material for: Machine learning forecasts suggest a concentration paradox in international student mobility to the United Kingdom
Source: Nat Commun. 2026 Sep 17;17:9604. doi: 10.1038/s41467-026-77425-z (PMC13586154; doi:10.1038/s41467-026-77425-z)
Supplement: Supplementary file 3 [file 41467_2026_77425_MOESM3_ESM.pdf]

nature portfolio

Peer Review File

Machine Learning Forecasts Suggest a Concentration Paradox in International Student Mobility to the United Kingdom

Corresponding Author: Dr Ruth Neville

This file contains all reviewer reports in order by version, followed by all author rebuttals in order by version.

Version 0:

Reviewer comments:

Reviewer #1

(Remarks to the Author)
This is an interesting and well-constructed study which applies machine learning techniques to the forecasting of international student inflows to the United Kingdom. A good case is made for the importance of this topic in the context of the considerable growth in international student mobility in higher education in recent years; the growing financial dependence of the UK higher education system on international student income; and the jeopardy brought to the latter by increased volatility in student inflows. In these circumstances, scholars, policymakers, university leaders and others have an interest in the adoption of more accurate and reliable forecasting tools. While the focus is on the UK, there are important lessons from the case for other countries.

The analysis compares three different techniques for forecasting. The latter of these uses a relatively novel - at least for this topic - machine learning technique of XGBoost. The three techniques are explained and the results compared. The dataset used is clearly set out and appropriate for the task, with a substantial time series. I am not an expert in the forecasting techniques utilised, particularly the machine learning approach, but I have sufficient grounding in quantitative analysis to have understanding of the basic implementations undertaken here.

The ML technique is demonstrated to give more accurate forecasts than ARIMA and gravity models, although the gains are not spectacular, and unsurprisingly none of the approaches handles significant structural shocks well, such as Brexit and the Covid-19 pandemic. Nevertheless, the substantive conclusion drawn about the 'concentration paradox' is very interesting and noteworthy, meaning that this could become an important reference for future research.

An issue which needs to be addressed is labelling of figures in the manuscript. Figure 4 and Figure 5 are mentioned in the text (p. 5) but there are no such figures presented, including in the supplementary material. There being figures 4 and 5 implies there should be a Figure 3 too, but that is neither mentioned nor displayed. This needs to be corrected.

(Remarks on code availability)

Reviewer #2

(Remarks to the Author)
This is an insightful study which makes use of UCAS data between 2010-2024 to forecast successful undergraduate applications through to 2030. By applying machine learning methods, this study finds an increasingly concentrated student pool from China, India, and the EU, which comprise of over 60% of applications by 2030. The paper lists three research objectives, 1. comparing predictions between the machine learning methods and traditional forecasting methods; 2. project future applications from origin countries to the UK; 3. document changes in the concentration of UK international student demand.

While the results look insightful and contributing to the literature, my main concern is there may be a mismatch between the stated research aim of study and the empirical measure used. Specifically, the paper clearly states that it investigates

international student mobility (ISM) to the UK, which sounds like using a direct measure of enrolments of international students in the UK, e.g., as reported in official statistics such as Higher Education Statistics Agency (HESA) data on international student enrolments, or student visa issues reported by home office statistics.

For example, one of the studies cited, British Council 2024, uses the measure of share of global outbound student which is based on UNESCO data for 2020; in another study, Office for Students 2024 seems to use HESA statistics on international student status. However, this study relies on UCAS undergraduate (only FTE?) application data, specifically what the paper refers to 'successful' applications.

Moreover, it does not seem to clearly define what the 'successful' application (acceptances) is. Is that application receiving offers (at least one)? with or without conditions? firm or insurance acceptances? Is that confirmed and followed by real enrolment. These application stages may have substantially different levels of commitment and are subject to varying degrees of attrition, particularly for international UCAS applicants.

Nevertheless, the implications of focusing on such 'successful' applications may also need further clarification. In particular, does the analysis imply that conversion rates from successful applications to enrolments are stable over time and countries? Does it assume that differences in the use of UCAS applications are also stable between EU and other international applicants over time (e.g., 95% of EU vs. 60% of non-EU as per UCAS 2019)? Or does it suggest that course competitiveness in application would be stable across time and international students? I am not familiar with UCAS data (definitely not an expert in using it), but these questions come to my mind in a general sense.

Altogether, this study has used a proxy of ISM rather than the direct measure of it, which would be fine for empirical research. However, given the nature of future predictions, along with its acknowledged uncertainties, projected trends in successful applications may not translate consistently into future international student enrolments and mobility, which limits the interpretation of the forecasts as indicators of ISM.

In addition, as a stated objective, I feel the comparisons between ARIMA, gravity, and XGBoost predictions should be included in the main text rather than in the supplementary information. Providing the detailed results in the main context would improve transparency and help readers assess the methodological contributions and performance of the different methods.

While the decision to combine students from (mainland) China and HK in the trend analysis is understandable, I think the result would require some additional justification or robustness checks (e.g., combining both student groups in the prediction models). Specifically, students from mainland China and HK may be considerably different in terms of study motivations, mobility outcomes, visa requirements, language proficiency, and post study plans.

In particular, HK students would be less subject to visa uncertainty, language and/or cultural barriers when studying in the UK, and their mobility outcomes may be more influenced by policy updates such as BNO visa arrangements. Presenting the combined student group for China mianland and HK may therefore overlook significant heterogeneity and potentially bias both the methodological design and the substantive conclusions.

As reported in the supplementary information, the normalised mean absolute error (NMAE) for mainland China predictions is 0.22 while only 0.06 for HK, which seem to be a significant difference. A robustness analysis combining these two groups for predictions, if data allows, would strengthen the robustness of the results.

In conclusion, despite these comments, the paper has a solid basis to make contributions. I think authors will be able to address the points raised in this review and resubmit a revised version.

(Remarks on code availability)

Version 1:

Reviewer comments:

Reviewer #1

(Remarks to the Author)
The revisions have fully addressed my comments.

(Remarks on code availability)

Reviewer #2

(Remarks to the Author)
Thanks for providing these revisions, I think the authors have addressed most of my concerns.

A few minor points for consideration:

I can see the rationale to do correlation analysis on the counts of undergraduate enrolments between UCAS and HESA, I was wondering if authors can extend the analysis to HESA all students, e.g., comparing UCAS undergraduate acceptances with HESA entrants for all full time and part time, undergraduates and postgraduate, given the research objectives on (all) international student mobility; likewise the validation check between annual UCAS acceptances and HESA Table 28 enrolment counts.

Another interesting point is the comparatively weaker correlation between UCAS acceptances and HESA undergraduate enrolment counts for HK (0.670, not entrant-specific), which seems to be a special case. I think a plausible explanation may relate to the treatment of domicile or permanent address within HESA data (Table 28 reports 'non-UK permanent address students'), e.g., If HK students come to the UK under BNO visa route, many may report a UK permanent address prior to study, even though they were originally international applicants through UCAS?

Lastly, a few typos need correction, e.g.,
1. Line 103 seems to omit Section 5 (conclusion)
2. Line 181 and 183, Table ??
3. Supplementary Line 195 as explored in [?]

(Remarks on code availability)

Version 2:

Reviewer comments:

Reviewer #2

(Remarks to the Author)
Thank you, I think authors have addressed all my concerns, recommend it for publication!

(Remarks on code availability)

Open Access This Peer Review File is licensed under a Creative Commons Attribution 4.0 International License, which permits use, sharing, adaptation, distribution and reproduction in any medium or format, as long as you give appropriate credit to the original author(s) and the source, provide a link to the Creative Commons license, and indicate if changes were made.
In cases where reviewers are anonymous, credit should be given to 'Anonymous Referee' and the source.
The images or other third party material in this Peer Review File are included in the article’s Creative Commons license,

unless indicated otherwise in a credit line to the material. If material is not included in the article's Creative Commons license and your intended use is not permitted by statutory regulation or exceeds the permitted use, you will need to obtain permission directly from the copyright holder.
To view a copy of this license, visit https://creativecommons.org/licenses/by/4.0/

Response to Reviewers

Machine Learning Forecasts Reveal a Concentration Paradox in UK International Student Mobility

Ruth Neville, Francisco Rowe, Emilio Zagheni

We thank the Reviewers for their careful reading of our manuscript and for their constructive and insightful suggestions. We have revised the manuscript and Supplementary Information accordingly. The revisions clarify the definition and interpretation of the UCAS acceptance data, add supplementary validation against public HESA undergraduate data, move key model comparison results into the main text, and provide additional explanation of the modelling framework and aggregation of forecasts for Mainland China and Hong Kong.

Key changes are as follows:

- Corrected figure numbering and cross-references throughout the manuscript and Supplementary Information.
- Added an explicit definition of UCAS acceptances in the Methods section (Section 6.1: Data and preprocessing) and clarified interpretation of forecasts as projections of confirmed placements within the UCAS admissions system.
- Added a supplementary validation exercise comparing UCAS acceptances with publicly available HESA undergraduate data, showing strong aggregate temporal alignment, strong cross-sectional correspondence in the composition of major non-EU origin countries, and strong origin-specific temporal correspondence for most major sending countries.
- Expanded discussion of limitations to clarify that forecasts of acceptances do not imply stable conversion rates to enrolment.
- Moved key model comparison results (ARIMA, gravity, and XGBoost) into the main text (Section 3.1: Model Performance).
- Added a new supplementary figure presenting separate origin-level forecast trajectories for Mainland China and Hong Kong (Supplementary Figure 2) and clarified that the combined series shown in the main figures is constructed through post-estimation aggregation.

Below we include each reviewer comment followed by a detailed response. All referenced changes have been incorporated into the revised manuscript and Supplementary Information. A tracked and untracked version of the manuscript and Supplementary Information is included in the resubmission.

Reviewer #1

Reviewer comment: This is an interesting and well-constructed study which applies machine learning techniques to the forecasting of international student inflows to the United Kingdom. A good case is made for the importance of this topic in the context of the considerable growth in international student mobility in higher education in recent years; the growing financial dependence of the UK higher education system on international student income; and the jeopardy brought to the latter by increased volatility in student inflows. In these circumstances, scholars, policymakers, university leaders and others have an interest in the adoption of more accurate and reliable forecasting tools. While the focus is on the UK, there are important lessons from the case for other countries.

We thank the reviewer for their positive and encouraging assessment of the manuscript and for recognising the potential contribution of this work to the literature on international student mobility and forecasting.

Reviewer comment: The analysis compares three different techniques for forecasting. The latter of these uses a relatively novel - at least for this topic - machine learning technique of XGBoost. The three techniques are explained and the results compared. The dataset used is clearly set out and appropriate for the task, with a substantial time series. I am not an expert in the forecasting techniques utilised, particularly the machine learning approach, but I have sufficient grounding in quantitative analysis to have understanding of the basic implementations undertaken here.

We thank the reviewer for their appreciation of the manuscript, and for highlighting that the methods are both clearly explained and accessible to a general audience with some background in quantitative analysis.

Reviewer comment: The ML technique is demonstrated to give more accurate forecasts than ARIMA and gravity models, although the gains are not spectacular, and unsurprisingly none of the approaches handles significant structural shocks well, such as Brexit and the Covid-19 pandemic. Nevertheless, the substantive conclusion drawn about the ‘concentration paradox’ is very interesting and noteworthy, meaning that this could become an important reference for future research.

We thank the reviewer for their appreciation of the relevance of the substantive results of the manuscript.

Reviewer comment: An issue which needs to be addressed is labelling of figures in the manuscript. Figure 4 and Figure 5 are mentioned in the text (p. 5) but there are no such figures presented,

including in the supplementary material. There being figures 4 and 5 implies there should be a Figure 3 too, but that is neither mentioned nor displayed. This needs to be corrected.

Response: Thank you for highlighting this issue. We have corrected all figure numbering and cross-references throughout the manuscript and Supplementary Information. In the revised manuscript, figures are now consistently numbered and all in-text references correspond to the appropriate figures. These corrections occur throughout the Results section and associated figure captions.

Reviewer #2

General remarks

We thank the reviewer for their thoughtful and constructive comments. In response, we have revised the manuscript to clarify the definition and interpretation of the UCAS acceptance data used in the analysis, improved the presentation of model comparisons, and clarified the treatment of Mainland China and Hong Kong in the forecasting framework.

Reviewer comment:

This is an insightful study which makes use of UCAS data between 2010-2024 to forecast successful undergraduate applications through to 2030. By applying machine learning methods, this study finds an increasingly concentrated student pool from China, India, and the EU, which comprise of over 60% of applications by 2030. The paper lists three research objectives, 1. comparing predictions between the machine learning methods and traditional forecasting methods; 2. project future applications from origin countries to the UK; 3. document changes in the concentration of UK international student demand.

We thank the reviewer for their positive assessment of the manuscript and for clearly summarising the objectives of the study.

Reviewer comment: While the results look insightful and contributing to the literature, my main concern is there may be a mismatch between the stated research aim of study and the empirical measure used. Specifically, the paper clearly states that it investigates international student mobility (ISM) to the UK, which sounds like using a direct measure of enrolments of international students in the UK, e.g., as reported in official statistics such as Higher Education Statistics Agency (HESA) data on international student enrolments, or student visa issues reported by Home Office statistics.

For example, one of the studies cited, British Council 2024, uses the measure of share of global outbound student which is based on UNESCO data for 2020; in another study, Office for Students 2024 seems to use HESA statistics on international student status. However, this study relies on

UCAS undergraduate application data, specifically what the paper refers to as 'successful' applications.

Moreover, it does not seem to clearly define what the 'successful' application (acceptances) is. Is that application receiving offers (at least one)? With or without conditions? Firm or insurance acceptances? Is that confirmed and followed by real enrolment? These application stages may have substantially different levels of commitment and are subject to varying degrees of attrition, particularly for international UCAS applicants.

Nevertheless, the implications of focusing on such 'successful' applications may also need further clarification. In particular, does the analysis imply that conversion rates from successful applications to enrolments are stable over time and across countries? Does it assume that differences in the use of UCAS applications are also stable between EU and other international applicants over time (e.g., 95% of EU vs. 60% of non-EU as per UCAS 2019)? Or does it suggest that course competitiveness in application would be stable across time and international students? I am not familiar with UCAS data (definitely not an expert in using it), but these questions come to mind in a general sense.

Altogether, this study has used a proxy of ISM rather than a direct measure. While this can be appropriate for empirical research, projected trends in successful applications may not translate consistently into future international student enrolments and mobility, which limits the interpretation of the forecasts as indicators of ISM.

Response: We thank the reviewer for these important comments. We agree that UCAS acceptances represent an administrative proxy rather than a direct measure of realised international student mobility, and also recognise that it is a good predictor of international student enrolment. To address the revirewer's concern, we have revised the manuscript in several places to clarify both the definition of the outcome variable, interpretation of the forecasts and compare UCAS acceptance and HESA enrolment data.

First, in the Methods section ("Data and preprocessing"), we now explicitly define UCAS acceptances as applicants who have accepted an offer of a place through the UCAS admissions system. This stage follows the submission of an application and the receipt of one or more conditional or unconditional offers and represents a confirmed admissions placement within the UCAS system. The revised definition appears in Section 6.1 (Data and preprocessing), lines 378–384. The revised text now reads:

> *The dependent variable is the annual count of successful undergraduate applications from each origin country. In UCAS terminology, an acceptance refers to an applicant who has firmly accepted an offer of a place at a UK higher education institution through the UCAS admissions system. This stage follows the submission of an application and the receipt of one or more conditional or unconditional offers. An acceptance therefore represents a confirmed admissions placement and a high-commitment stage in the mobility decision process, although final enrolment remains contingent on meeting offer conditions and completing institutional registration.*

Second, we clarify that the forecasts presented in this study should be interpreted as projections

of international undergraduate acceptances within the UCAS system rather than guaranteed realised enrolments or visa issuances. This clarification is now stated explicitly in the Methods section (Section 6.1, lines 384–394) and reiterated in the Discussion (lines 325–344). The revised text reads:

> *While not all accepted applicants ultimately enrol, UCAS acceptance data provide the most temporally granular and up-to-date national-level administrative measure of international undergraduate admissions outcomes available. UCAS acceptance data become available within the same academic year enabling timely predictions of international mobility flows into the UK. Alternative national datasets, such as Higher Education Statistics Agency (HESA) enrolment data, are typically released with a lag of one to two years, limiting their suitability for near-term forecasting. As the centralised admissions platform for UK undergraduate education, UCAS provides a consistent longitudinal record of international student admissions across origin countries. We therefore interpret accepted applications as an administrative proxy for international student demand and mobility, while recognising that they may not perfectly correspond to final enrolment counts. The forecasting models therefore predict accepted undergraduate placements within the UCAS admissions system rather than realised international student enrolments or visa issuances.*

Third, to assess the extent to which this proxy tracks broader realised undergraduate inflows, we conducted a series of validation exercises comparing UCAS acceptances with publicly available HESA undergraduate data. We compared UCAS acceptances with HESA undergraduate entrant counts for EU and Non-EU domicile groups and found very strong temporal alignment (Pearson $r = 0.998$ and $r = 0.975$, respectively). We further examined cross-sectional composition by comparing the distribution of major non-EU origin countries in UCAS acceptances and HESA undergraduate enrolments, finding similarly strong correspondence ($r = 0.978$). Additionally, we assessed origin-specific temporal dynamics using publicly available HESA country-level undergraduate data over 2014–2024, showing strong correlations for most major origin countries (e.g. China $r = 0.973$, India $r = 0.984$), with some heterogeneity across origins. Together, these validation tests indicate that UCAS acceptances capture both temporal dynamics and cross-country composition of international undergraduate mobility, despite differences in administrative definition and coverage. Full details are provided in the Supplementary Information (Section S5).

Fourth, we expand the discussion of limitations to emphasise that conversion rates from acceptances to enrolments may vary across countries and over time, and that changes in application channels, UCAS coverage, or institutional selection processes could affect the relationship between acceptances and realised enrolments. These clarifications appear in the Limitations subsection of the Discussion (lines 325–344). The revised text now reads:

> *We focus on undergraduate applications captured via UCAS. Our analysis relies on UCAS accepted applications rather than realised enrolments. While UCAS captures the majority of international undergraduate entrants and provides the most consistent and temporally granular administrative measure available across origin countries and time, conversion rates from acceptance to enrolment may vary across countries, subjects, and contexts. Forecasts should therefore be interpreted as projections of international student demand and confirmed undergraduate placements rather than realised enrolments.*

Finally, we clarify the advantages of UCAS data for forecasting analysis. As the centralised admissions platform for UK undergraduate education, UCAS provides a consistent country-year administrative record of international undergraduate admissions outcomes across origin countries. In addition, UCAS data are released on an annual cycle with minimal publication lags following the admissions cycle (e.g. we were able to access 2024/25 data immediately at the end of the 2024 admissions cycle), whereas alternative national datasets such as Higher Education Statistics Agency (HESA) enrolment data are typically released with a lag of one to two years (2024/25 data only became available in January 2026).

UCAS data also provide a longer and more consistent historical time series for country-level undergraduate admissions than is readily available from published HESA outputs, enabling the construction of extended country-level panels required for forecasting. Publicly available HESA data are valuable for validation but are either more limited in temporal coverage or not available at the same country-year resolution. These characteristics make UCAS acceptances well suited for modelling and forecasting near-term dynamics in international undergraduate demand. We clarify this rationale in Section 6.1 (Data and preprocessing) and in the Discussion.

Together, these revisions clarify both the administrative definition of the outcome variable and the appropriate interpretation of the forecasts, and demonstrate that UCAS acceptances provide a consistent and empirically supported proxy for international undergraduate mobility patterns.

Reviewer comment: In addition, as a stated objective, I feel the comparisons between ARIMA, gravity, and XGBoost predictions should be included in the main text rather than in the supplementary information. Providing the detailed results in the main context would improve transparency and help readers assess the methodological contributions and performance of the different methods.

Response: We agree with this suggestion and have revised the manuscript accordingly. In the revised manuscript, the comparison between ARIMA, negative binomial gravity, and XGBoost models is now presented in the main text Results Section 3.1 ("Model Performance"). This section includes both the aggregate performance metrics and the predicted-versus-observed comparison figure (Figure 1). Extended diagnostics and additional country-level evaluation results remain available in the Supplementary Information.

Reviewer comment: While the decision to combine students from (mainland) China and HK in the trend analysis is understandable, I think the result would require some additional justification or robustness checks (e.g., combining both student groups in the prediction models). Specifically, students from mainland China and HK may be considerably different in terms of study motivations, mobility outcomes, visa requirements, language proficiency, and post study plans.

In particular, HK students would be less subject to visa uncertainty, language and/or cultural barriers when studying in the UK, and their mobility outcomes may be more influenced by policy updates such as BNO visa arrangements. Presenting the combined student group for China

mainland and HK may therefore overlook significant heterogeneity and potentially bias both the methodological design and the substantive conclusions.

As reported in the supplementary information, the normalised mean absolute error (NMAE) for mainland China predictions is 0.22 while only 0.06 for HK, which seem to be a significant difference. A robustness analysis combining these two groups for predictions, if data allows, would strengthen the robustness of the results.

Response: We thank the reviewer for raising this important point. We agree that Mainland China and Hong Kong represent distinct origin contexts with different policy environments and mobility dynamics. In the modelling framework used in this study, Mainland China and Hong Kong are treated as separate origin systems throughout model estimation and forecasting. This modelling framework allows origin-specific dynamics to be captured directly, ensuring that differences in policy exposure, mobility behaviour, and volatility between Mainland China and Hong Kong are reflected in the estimates.

Specifically, all models are estimated at the origin–year level, meaning that predictions are generated independently for each origin country. The combined "China and Hong Kong" series shown in the main figures is therefore constructed post-estimation by aggregating the origin-specific forecasts for presentation purposes. This aggregation does not affect the estimation of the models themselves.

To clarify this point, we have revised the manuscript and Supplementary Information to explicitly describe the modelling and aggregation approach. In addition, we provide a supplementary figure (Supplementary Figure 2) presenting separate origin-level forecast trajectories for Mainland China and Hong Kong alongside the aggregated series used in the main text. These additions demonstrate the distinct dynamics and uncertainty ranges associated with the two origin systems.

The relevant clarification now appears in the Supplementary Information (Section S4.2). The revised text reads:

> *Mainland China and Hong Kong are treated as distinct origin systems throughout model estimation and forecasting. All models are estimated at the origin–year level, and aggregated values shown in the main figures are constructed by summing origin-specific forecasts rather than through pooled or joint estimation.*
>
> *Given substantive differences in policy exposure and mobility dynamics between Mainland China and Hong Kong, we present separate origin-level forecasts here to demonstrate distinct trajectories and uncertainty ranges. Differences in predictive error between Mainland China (NMAE = 0.22) and Hong Kong (NMAE = 0.06) reflect differences in scale and volatility rather than joint estimation, as each origin is modelled independently.*

Additionally, we add further clarification to the results when reporting the results for Mainland China and Hong Kong (Section 3.2).

> *Although presented here as a combined series for interpretability, forecasts are estimated independently for Mainland China and Hong Kong and aggregated post-estimation. Separate origin-level*

trajectories (Supplementary Fig. S2) indicate differences in scale and volatility between the two systems, with the decline in applications more pronounced for Mainland China than Hong Kong, but the combined trajectory remains dominated by Mainland China due to its substantially larger application volume.

These revisions clarify that Mainland China and Hong Kong are not pooled during model estimation and that the combined series shown in the main figures represents a post-estimation aggregation used for presentation. The supplementary analysis further demonstrates the heterogeneity between the two origin systems. We trust that this clarification addresses the reviewer's concern.

Finally, we note that because Mainland China and Hong Kong are already modelled independently, combining them during model estimation would not provide an additional robustness check and could instead obscure meaningful heterogeneity between the two origin systems. The modelling framework adopted here therefore preserves origin-specific dynamics while allowing aggregated presentation for interpretability in the main figures.

We thank the reviewers again for their thoughtful and constructive feedback, which has helped improve the clarity and robustness of the manuscript.

Response to Reviewers: Second Revision

Machine Learning Forecasts Reveal a Concentration Paradox in UK International Student Mobility

Ruth Neville, Francisco Rowe, Emilio Zagheni

We thank the Reviewers for their continued engagement with the manuscript and for their constructive comments on the revised version. In this second revision, we have clarified the empirical scope of the paper as undergraduate international student mobility through the UCAS admissions system, extended the UCAS–HESA validation to include HESA all-student enrolment counts, added a specific discussion of Hong Kong as a special case in the validation results, and corrected the remaining unresolved cross-references and typographical issues.

Reviewer #1

Reviewer comment: The revisions have fully addressed my comments.

Response: We thank the reviewer for their positive assessment of the revised manuscript. We are grateful for their constructive comments, which helped us improve the clarity and presentation of the paper.

Reviewer #2

Reviewer comment: Thanks for providing these revisions, I think the authors have addressed most of my concerns.

Response: We thank the reviewer for their constructive assessment of the revised manuscript and for the additional suggestions, which have helped us further clarify the empirical scope of the study and strengthen the UCAS–HESA validation checks.

Reviewer comment: I can see the rationale to do correlation analysis on the counts of undergraduate enrolments between UCAS and HESA, I was wondering if authors can extend the analysis to HESA all students, e.g., comparing UCAS undergraduate acceptances with HESA entrants for all full time and part time, undergraduates and postgraduate, given the research objectives on (all) international student mobility; likewise the validation check between annual UCAS acceptances and HESA Table 28 enrolment counts.

Response: We thank the reviewer for this helpful suggestion. In response, we have extended the UCAS–HESA validation exercise to include a broader comparison with all-student enrolment counts

from HESA's public country-of-domicile table: Table 28, "Non-UK domiciled HE students by HE provider and country of domicile, 2014/15 onward". Specifically, we compared annual UCAS undergraduate acceptances with HESA non-UK permanent address student counts aggregated across all levels and modes of study, including undergraduate and postgraduate students, full-time and part-time modes, and both new and continuing enrolments.

We note that this is not a like-for-like comparison. UCAS acceptances capture undergraduate admissions flows, whereas the HESA all-student measure captures broader enrolment stocks across study levels and modes. Nevertheless, the additional sensitivity analysis shows strong temporal alignment for most major origins, including China ($r = 0.957$), India ($r = 0.991$), Malaysia ($r = 0.940$), Nigeria ($r = 0.951$), the United Arab Emirates ($r = 0.970$), and the United States of America ($r = 0.953$). The 2024 selected-origin composition correlation is lower for the all-student HESA comparison ($r = 0.787$) than for the undergraduate-only HESA comparison ($r = 0.978$), which is expected given that the all-student measure includes postgraduate students, part-time modes, and continuing enrolments outside the UCAS undergraduate admissions process.

We have added this analysis to Supplementary Section S5.3 and report the results in Supplementary Table 6. We have also clarified in the main manuscript, in the aims and in the text, that the empirical focus of the paper is undergraduate international student mobility through the UK admissions system, rather than all international student enrolments across all study levels and modes.

Reviewer comment: Another interesting point is the comparatively weaker correlation between UCAS acceptances and HESA undergraduate enrolment counts for HK (0.670, not entrant-specific), which seems to be a special case. I think a plausible explanation may relate to the treatment of domicile or permanent address within HESA data (Table 28 reports 'non-UK permanent address students'), e.g., If HK students come to the UK under BNO visa route, many may report a UK permanent address prior to study, even though they were originally international applicants through UCAS?

Response: We agree that Hong Kong is a special case and thank the reviewer for suggesting this explanation. The extended comparison with HESA's public country-of-domicile table reinforces this interpretation. While Hong Kong showed a weaker correlation in the undergraduate HESA comparison ($r = 0.670$), the association is weaker still in the broader all-student HESA comparison ($r = 0.366$). We have added text noting that this may partly reflect differences between UCAS and HESA country classifications. UCAS records applicant domicile at the admissions stage, whereas HESA's public country-of-domicile table reports students by non-UK permanent address. Following the introduction of the British National Overseas route, some Hong Kong-origin students may have reported, or later come to report, a UK permanent address by the time of enrolment or continued study, reducing their visibility in HESA's Hong Kong non-UK permanent-address counts.

We present this as an interpretation and limitation of cross-source validation. This clarification has been added to Supplementary Section S5.4 and briefly noted in the limitations section of the

main manuscript.

Reviewer comment: Lastly, a few typos need correction, e.g., 1. Line 103 seems to omit Section 5 (conclusion) 2. Line 181 and 183, Table ?? 3. Supplementary Line 195 as explored in [?]

Response: We thank the reviewer for identifying these issues. We have corrected the manuscript structure sentence so that it explicitly refers to Section 5 as the conclusion. We have also replaced unresolved cross-references to supplementary tables in the main manuscript with manually specified Supplementary Table references where the main manuscript and Supplementary Information are compiled separately. Finally, we corrected the unresolved citation in the Supplementary Information and checked the revised manuscript and Supplementary Information for remaining unresolved references or citations.

We thank the reviewers again for their thoughtful and constructive feedback, which has helped improve the clarity and robustness of the manuscript.
